# Graphene Photodetectors Toward Practical Technologies for Biosensing: Mechanisms, Architectures, Applications, and Integration Challenges

**DOI:** 10.3390/bios16100536

**Published:** 2026-09-24

**Authors:** Xinzhuo Sun, Hao Su, Zhongjie Zhu, Dandan Sang, Zhanfeng Zhang, Hang Li, Santosh Kumar, Qinglin Wang

**Affiliations:** 1 School of Physics Science and Information Technology, Liaocheng University, Liaocheng 252000, China; 2024407142@stu.lcu.edu.cn (X.S.); 2023404864@stu.lcu.edu.cn (H.S.); 2 Shandong Liaocheng Laixin Powder Materials Science and Technology Co., Ltd., Liaocheng 252000, China; 15063550854@163.com; 3 Shandong Jinhengli Mechanical Manufacturing Co., Ltd., Taian 271200, China; 13345290998@163.com (Z.Z.); 18653802835@163.com (H.L.); 4 Centre of Excellence for Nanotechnology, Department of Electronics and Communication Engineering, Koneru Lakshmaiah Education Foundation, Vaddeswaram 522302, Andhra Pradesh, India; santosh@kluniversity.in

**Keywords:** graphene photodetectors, two-dimensional materials, broadband photodetection, device engineering, intelligent sensing

## Abstract

Graphene, with its 2D structure, zero bandgap, high carrier mobility, and broadband absorption, is an ideal platform for next-generation photodetectors with substantial promise for biosensing applications. This review summarizes recent progress in graphene photodetectors along the following two lines: device physics and applications. At the device level, the following three types are discussed: heterojunction, photogating, and photothermoelectric/plasmonic. Performance enhancements via band engineering, interface control, and nanostructure design are revealed. For example, a SiO_2_/CuO buffer layer in heterojunction detectors achieves an on/off ratio of 1.1 × 10^2^, greatly improving responsivity. At the application level, breakthroughs and challenges in extreme environment monitoring, flexible wearables, large-scale integration, multifunctional intelligent sensing, and full-band detection are explored. These advances show broad prospects in biomedical sensing, wearable health monitoring, intelligent imaging, high-speed optical communication, and defense. This review constructs a knowledge graph covering physical mechanisms, material processes, and system applications. It analyzes core contradictions in performance trade-offs, process compatibility, and system integration and outlines the following future directions: exploring new physical mechanisms, CMOS-compatible heterogeneous integration, and intelligent sensing memory computation integration. The goal is to provide theoretical and technical guidance for transitioning graphene photodetectors from the lab to practical applications, including biomedical diagnostics and wearable healthcare systems.

## 1. Introduction

As core sensing components of modern information technology, photodetectors play an irreplaceable role in fields such as optical communication, imaging, spectral analysis, extreme environment monitoring, and national defense security (Figure 1). As applications continue to expand towards scenarios that demand high speed, a wide spectrum, low power consumption, flexibility, and intelligence, traditional semiconductor detectors, owing to their inherent material properties, are gradually facing bottlenecks in terms of detection bands, response speed, mechanical flexibility, and integration compatibility. For instance, the response of silicon-based detectors drops sharply in the near-infrared and above bands, whereas many narrow-bandgap semiconductors, although capable of covering the infrared band, struggle to achieve both high performance at room temperature and low-cost integration [1]. Therefore, the development of a new generation of photoelectric detection materials and devices that feature wide spectral responses, high sensitivity, rapid responses, and excellent environmental adaptability has become a cutting-edge topic in both academic and industrial circles.

Against the above backdrop, graphene, as a typical two-dimensional material, offers a revolutionary material platform for breaking through the performance limitations of traditional detectors with its unique zero-bandgap band structure, extremely high carrier mobility, excellent mechanical flexibility, and broad-spectrum light absorption characteristics spanning from ultraviolet to terahertz [2,3]. This assertion is well-grounded when graphene is compared with alternative materials. Bulk graphite and conventional carbon materials, for instance, lack the electronic bandgap tunability and mechanical compatibility with ultrathin devices that graphene readily provides. Carbon nanotubes, while promising, are hampered by chirality-dependent bandgaps and contact resistance non-uniformities, whereas graphene offers a planar, defect-free surface amenable to large-area integration. Other emerging two-dimensional semiconductors such as transition metal dichalcogenides and black phosphorus each possess their own merits, yet neither matches graphene’s combination of ultra-broadband absorption, ultrahigh carrier mobility, and robust ambient stability, the latter being a particular weakness of black phosphorus. It is this distinctive synergy of properties that elevates graphene above its competitors and underpins its status as a uniquely versatile platform for next-generation photodetection. In recent years, researchers have achieved high responsiveness and low dark current detection in the mid-infrared band by constructing heterojunctions with graphene and semiconductor materials [1] or by integrating strongly absorbing materials such as perovskite quantum dots, in which an astonishing responsivity of up to 8.2 × 10^8^ A/W was achieved in the visible light band [4]. These achievements demonstrate that through core device strategies such as heterojunction engineering, optical gate effect regulation, photothermal electrics, and plasma enhancement, the performance boundaries of graphene photodetectors are continuously expanding and gradually advancing into cutting-edge fields such as flexible wearables and intelligent sensing.

The realization of these performance breakthroughs relies critically on material preparation and device fabrication methods, as briefly outlined below. Currently, the large-scale preparation of high-quality graphene mainly relies on the chemical vapor deposition (CVD) method. The typical process is to grow single-layer graphene on a transition metal as a catalytic substrate at approximately 1000 °C, and then transfer it to the target substrate by the wet method. To meet the requirements of flexible electronics for low-temperature processes, plasma-enhanced chemical vapor deposition (PECVD) technology can be used to directly grow graphene at approximately 400 °C, but the crystal quality of the product still needs to be optimized. In addition, the SiC thermal decomposition method and the reduction in graphene oxide (rGO) solution method are respectively suitable for high-quality quasi-free-standing graphene and low-cost large-area coating scenarios. At the device construction level, the wet transfer method is the mainstream approach, but it has problems of polymer residue and interface contamination; direct growth without transfer and van der Waals hetero-integration represent important directions for improving interface quality and process compatibility. In terms of characterization, Raman spectroscopy is the core tool for determining the number of graphene layers and defect density, atomic force microscopy is used for thickness confirmation, and electrical transport measurement and photoelectric performance testing directly evaluate key parameters such as the carrier mobility, response rate, and detection rate of the devices. The above preparation and characterization methods are the basis for understanding the subsequent design logic and performance optimization of various devices, and the relevant details will be elaborated in each section with specific cases.

However, despite the fruitful achievements, this field still faces multiple challenges and deep-seated contradictions when moving towards practical applications. First, the inherent trade-offs among performance indicators are prominent. For instance, although the graphene ultraviolet detector integrated with GaN nanoparticles achieved an ultra-high responsivity of 200.69 A/W, its response time was at the millisecond level. This contradiction stems from the gain mechanism: the longer the trap binds the carriers, the higher the gain, but the release process is also slower, thereby limiting the response speed [5]. Second, the bottlenecks in the process and integration are significant. Although technologies such as 400 °C low-temperature plasma-enhanced chemical vapor deposition that are more compatible with back-end processes have emerged [2], most high-performance heterojunctions still rely on the wet transfer of graphene. It is difficult to ensure large-area uniformity and high yield with this process, and the interface is easily affected by the environment [4]. Furthermore, the verification and integration capabilities for system-level applications are insufficient. For instance, in high-end applications such as mid-infrared imaging, the comprehensive performance of the device in terms of array integration and lower noise still needs to be improved.

In view of this, this study is organized along two main threads to systematically address the key challenges in transitioning graphene photodetectors from laboratory research to practical applications. The first thread focuses on the three core device architectures, namely the heterojunction, photogating, and photothermoelectric/plasmonic types, with an emphasis on their working mechanisms, structural configurations, and performance optimization strategies. The second thread examines the performance breakthroughs achieved by these devices across four major application frontiers, including extreme environment monitoring, flexible wearables and large-scale integration, multifunctional intelligent sensing, and full-band detection from ultraviolet to terahertz. Through this dual-thread framework, this review constructs a knowledge graph that bridges device physics, materials engineering, and system-level applications, providing both a fundamental understanding and a practical roadmap for the future development of graphene-based photodetection technologies [1,4,5,6].

## 2. Types of Graphene Photodetectors

With the development of photoelectric detection towards high speed, a wide spectrum, low power consumption, flexibility, and intelligence, traditional semiconductor detectors are constrained by the intrinsic characteristics of the materials, and the bottlenecks in the detection bands, response speed, mechanical flexibility, and integration compatibility are becoming increasingly prominent. Graphene, as a typical two-dimensional material, has become a revolutionary material platform that breaks through the performance limitations of traditional devices, owing to its zero-bandgap structure, ultra-high carrier mobility, excellent mechanical flexibility, and wide-spectrum light response characteristics spanning from ultraviolet to terahertz. There are presently the following three core technical types of photodetectors developed based on graphene, which mainly form three core technical systems: heterojunction photodetectors, photogate effect photodetectors, and photothermal-electric/plasma-enhanced photodetectors. Each achieves performance breakthroughs through unique working mechanisms and demonstrates different application advantages. The core idea of heterojunction photodetectors is to combine graphene with various semiconductor materials such as ZnO, silicon, quantum dots, and transition metal sulfides to construct a composite device that can synergistically leverage the advantages of both. It utilizes the high-efficiency light absorption capacity of semiconductor materials to compensate for the weak light absorption of graphene, while also taking advantage of the high-mobility transport channel of graphene. Thus, it demonstrates great potential in aspects such as wide spectral responses, high sensitivity, and fast responses. The working mechanism of the photogate effect-type photodetector relies on the trapping of photogenerated carriers at the interface to modulate the conductance of the graphene channel. The local electric field formed after the photogenerated carriers are captured by the local state generates the gate control effect, achieving the amplification of the channel current. This mechanism is regarded as one of the key paths to achieving ultra-high photoelectric gain, especially suitable for the sensitive detection of weak optical signals. The photothermal–electric/plasma-enhanced photodetector utilizes the photothermal–electric effect to achieve the separation of photogenerated carriers at zero bias voltage. That is, the temperature gradient produced by light drives the diffusion of carriers to form a photovoltage. At the same time, it significantly enhances the interaction between light and graphene through the nano-plasma structure and breaks through the intrinsic absorption limit of graphene through the local field enhancement effect. Thus, breakthroughs have been made in response speed, working bandwidth, and power consumption control. This chapter will systematically review the working principles, the latest research progress, core challenges faced, and future development directions of these three types of graphene photodetectors.

Before delving into the three types of devices, it is necessary to clarify the two different roles that graphene plays in photodetectors. Firstly, as a zero-bandgap light absorption layer/channel material, graphene, with its zero-bandgap energy band structure, can achieve broadband light absorption from ultraviolet to terahertz. However, the intrinsic absorption rate of a single-layer graphene is only about 2.3% and the zero-bandgap leads to a high carrier recombination rate, which typically limits the response rates of pure graphene detectors to typically a few mA/W level. Therefore, researchers have enhanced graphene’s performance as a light absorption layer by constructing graphene nanoribbons, introducing defect engineering, or combining it with other light-absorbing materials. Secondly, given graphene’s excellent conductivity and high transmittance, it can serve as an efficient transparent electrode and charge transport layer, forming heterojunctions with traditional light absorption materials. In this role, light absorption is mainly accomplished by composite semiconductor materials, while graphene is responsible for quickly collecting and transporting photogenerated carriers. The design strategies and performance optimization paths corresponding to these two roles in the devices are fundamentally different: the former focuses on enhancing light absorption and bandgap engineering, while the latter focuses on interface engineering and charge transfer efficiency optimization. Among the three types of devices discussed in this review, heterojunction-type devices usually involve the synergy of the two roles, while photogating-type and photovoltaic/plasmonic-type devices are more focused on the intrinsic characteristics of graphene as a channel material.

### 2.1. Heterojunction-Type Photodetector

Graphene is regarded as an ideal photoelectric material owing to its zero-bandgap structure and extremely high carrier mobility. However, its inherent weak light absorption property limits the performance of pure graphene detectors. To overcome this limitation, heterojunction engineering has become a key technical path. By combining graphene with various semiconductor materials, composite devices that can synergistically leverage the advantages of both sides are constructed, demonstrating great potential in terms of wide spectral responses, high sensitivity, and rapid responses. The heterojunction structures mainly include composite systems formed by graphene and one- and two-dimensional semiconductors. In these structures, graphene and low-dimensional semiconductors jointly participate in light absorption and charge separation, functioning synergistically. It is worth noting that heterojunctions formed by graphene and three-dimensional bulk semiconductors have also been widely studied. In such structures, light absorption is mainly accomplished by the bulk semiconductor, while graphene mainly serves as a transparent electrode or a carrier transport layer. For completeness, some representative work on three-dimensional heterojunctions is also cited, but its core functional positioning should be understood as that of an electrode/transistor layer role.

Interface engineering, particularly the modulation of contact properties and charge transfer behavior, has been extensively explored as a key strategy for optimizing heterojunction performance. Early research laid an important foundation for the design of heterojunction devices. Some studies have synthesized ZnO nanoparticles by pulsed laser fragmentation in liquid and integrated them with graphene, achieving high-response ultraviolet detection. This method provides a simple, efficient, and ligand-free approach for nanoparticle synthesis [7]. The regulation of interface characteristics has been proven to be crucial. By precisely adjusting the doping concentration difference between the graphene channel and the contact region through annealing processes at different temperatures, the junction barrier height and the built-in electric field intensity can be effectively regulated. This discovery has deepened the understanding of the interface charge transfer mechanism [8]. In addition, it has been proven that soaking the grown ZnO films in H_2_O_2_ solution at room temperature for simple surface treatment can effectively modify the surface state of ZnO, thereby transforming the graphene/ZnO interface from ohmic contact to stable Schottky contact. This provides a simple and efficient process solution for achieving rapid and low-power self-powered photovoltaic detection [9]. Beyond oxide-based systems, chemical doping has been shown to be an effective means by which to systematically enhance device performance. Directional chemical doping on graphene can be performed to precisely regulate its Fermi level, thereby significantly improving the Schottky barrier characteristics of graphene/germanium near-infrared detector devices. Experiments show that this method can simultaneously enhance responsivity and suppress dark current [10]. Another important interface control strategy is to introduce ultrathin interface modification layers, such as HfOx films, which can effectively increase the Schottky barrier height of graphene/silicon heterojunctions and promote the transport of photogenerated carriers by utilizing the oxygen vacancy channels within them, thereby significantly suppressing dark currents while maintaining high light response [11].

Beyond interface optimization, the incorporation of diverse functional materials has substantially enriched the material toolkit for graphene-based heterojunctions. The combination of graphene and quantum dots forms a class of highly promising van der Waals heterojunction systems. The strong light absorption and tunable bandgap characteristics of quantum dots, when combined with the high mobility of graphene, significantly enhance the detection performance, enabling such devices to exhibit extremely high responsivity potential across a wide spectral range from ultraviolet to infrared [12]. In addition, innovative physical mechanisms of devices have also been actively explored. For instance, the pyroelectric effect of ferroelectric materials is utilized to induce the formation of localized p-n junctions in graphene under temperature changes caused by light exposure. This research provides an important implementation approach for high-performance, wide-spectrum, high-sensitivity photodetection and next-generation optoelectronic devices that integrate two-dimensional materials with ferroelectric materials [13]. Innovations at the material level are equally remarkable. The emergence of semiconductor graphene nanoribbons has addressed the key issue of the zero band gap in graphene. Heterojunction detectors constructed with these nanoribbons as intrinsic light absorption layers have achieved outstanding comprehensive performance, marking the expansion of graphene’s application in optoelectronic devices from a simple transport layer to a functional absorption layer [14]. These studies indicate that the combination of graphene with low-dimensional nanomaterials such as quantum dots and nanoribbons has become a key nanoengineering strategy for enhancing light absorption and expanding the detection band from the material’s source.

Beyond the exploration of material systems, the fundamental understanding of heterojunction behavior has been substantially advanced by integrating theoretical simulations with advanced in situ characterization techniques. Density functional theory (DFT) calculations, for instance, have been employed to probe the electronic structure and interfacial charge transfer dynamics at heterojunctions. Combined with in situ infrared spectroscopy, DFT calculations have revealed the atomic-scale deprotonation and desorption processes occurring at perovskite surfaces during the atomic layer deposition of SnO_2_, providing critical insights into the interfacial chemistry that governs heterojunction performance [15]. In parallel, in situ Raman spectroscopy has proven invaluable for monitoring structural evolution during device fabrication. When coupled with machine learning algorithms, in situ Raman can identify the lowest critical phase transition temperatures with high precision, enabling the real-time optimization of thermal annealing processes for phototransistors [16]. Furthermore, advanced transmission electron microscopy (TEM) observations have been instrumental in linking nanoscale heterojunction architecture to macroscopic device characteristics. TEM analysis of flower-like hierarchical heterostructures has demonstrated that the unique grain boundary barriers and electronic properties arising from such morphologies play a critical role in suppressing thermal drift and enhancing sensitivity [17]. These theoretical and characterization studies, covering DFT calculations, in situ spectroscopy, and high-resolution microscopy, have not only deepened the mechanistic understanding of heterojunction photodetectors but also provided a robust foundation for the rational design of next-generation devices.

In recent years, significant progress has been made in the innovation of device structures and performance optimization. To maximize the built-in electric field for self-powered operation, asymmetric electrode designs have been adopted in van der Waals heterojunction detectors, such as the Au-InSe-Gr device (Figure 2a). This configuration leverages the work function difference between Au and graphene relative to InSe to form unequal Schottky barriers and a built-in electric field (Figure 2b); the IV characteristic of the Au-InSe-Gr heterojunction reveals a distinct rectifying behavior (Figure 2c), featuring a dark current as low as 0.1 nA at −1 V. Such an exceptionally low dark current not only confirms the effective noise suppression of the device but also serves as a critical prerequisite for ensuring a high signal-to-noise ratio and excellent specific detectivity in graphene-based photodetectors [18]. Another type of structure aimed at enhancing the interfacial electric field and carrier separation efficiency combines graphene with vertically arranged semiconductor nanowire arrays. This three-dimensional heterojunction structure provides a vast contact area and a fast charge collection path along the nanowire axis, thereby achieving an ultrafast photoelectric response [19]. This idea of combining graphene with customized nanostructures such as one-dimensional nanowires and three-dimensional nanowalls provides an effective way to break through the photoelectric performance bottleneck of traditional planar devices by constructing huge heterogeneous interfaces and directional charge channels. In the near-infrared band, by using advanced heteroepitaxy technology to integrate graphene with complex PN-type compound semiconductors on a single chip, detectors with both high responsiveness and high speed have been developed, such as graphene/InSb/AlSb heterointegrated photodetectors, demonstrating their application prospects in the field of optical communication (Figure 2d) [20]. For thin-film devices, buffer layer engineering has been systematically studied to optimize the interface charge behavior. Research shows that introducing a buffer layer made of specific materials between ZnO thin films and substrates can effectively regulate the interface band structure and carrier recombination dynamics. Thus, an enhanced on/off ratio (I_photo_/I_dark_ = 1.1 × 10^2^) and accelerated decay dynamics are achieved [21]. In addition, not only does the van der Waals heterocombination of graphene and novel two-dimensional semiconductors demonstrate outstanding performance in wideband self-powered detection, but also the initially integrated micro-wire array shows near-infrared imaging capabilities (Figure 2e) [22].

The exploration of multifunctionality and new device architectures is constantly pushing the boundaries of this field. A dual-wavelength detector capable of simultaneously responding to two independent bands, ultraviolet and infrared, has been successfully developed. This device based on a graphene/GaN heterojunction, through ingenious band engineering design, achieves the selective detection of specific spectral windows, providing a solution for the developing compact multispectral detection systems [23]. The organic–inorganic hybrid system also demonstrates unique advantages. The heterojunction constructed based on a single-crystal perovskite and graphene has successfully achieved rapid self-powered electro-optical detection. Its fast response and high detection performance have application potential in the field of low-power optical sensing and communication [24]. In terms of the diversification of device structures, the Schottky structure using graphene-doped polymers as the interface layer has been proven to effectively improve the performance of silicon-based heterojunctions. This structure combines the simplicity of fabrication with good photoconductive properties, such as the PVA/p-Si Schottky structure photodetector. A heterojunction is formed between P-type silicon and the graphene-doped PVA layer. The energy band alignment governs the barrier height and carrier transport (Figure 3a), with respect to the measurement system and device configuration (Figure 3b). Graphene incorporation facilitates charge transfer and improves interface quality, enhancing device performance [25]. A heterojunction was formed by using P-type graphene nanowalls and N-type silicon, with hafnium oxide deposited and grown in atomic layers inserted in the middle as the interface layer (Figure 3c). Graphene nanowalls (GNWs) are directly grown on silicon substrates through low-power remote plasma-enhanced chemical vapor deposition (Figure 3d), demonstrating a high-performance self-powered photodetector that does not require complex transfer processes [26]. This work highlights the significant value of in situ-grown nanostructures in avoiding transfer damage, improving interface quality and device uniformity, and is beneficial exploration for promoting the practical application of heterojunction graphene detectors. Earlier exploratory work also contributed important insights. For instance, by combining a three-dimensional vertical graphene network structure with floating indium tin oxide transparent channels, the optical path was prolonged and the carrier transport distance was significantly shortened, effectively enhancing the efficiency of light capture and carrier collection [27]. The strategy of constructing graphene–quantum dot–nanorods multi-heterojunctions has achieved efficient visible light detection through a cascaded charge separation process. Theoretical simulation research provides indispensable guidance for device design [28] and performance optimization, including the comprehensive simulation of the photoelectric characteristics of multilayer graphene and environmentally friendly semiconductor heterojunctions [29], as well as cutting-edge modeling and analysis of the photothermal ion detection mechanism based on graphene/silicon Schottky junctions [30]. Furthermore, extensive research also covers MIS broadband detector structures based on graphene/silicon nitride/silicon (Figure 3e) [31], low-temperature processes for the in situ preparation of TiO_2_-modified layers through oxygen plasma treatment [32], methods for precisely regulating the barrier height by chemical doping with polymer interface layers [33], and the in-depth exploration of the influence of environmental adsorbents on the properties of graphene/silicon Schottky junctions. There is also research on the mechanism of sound [34], an all-fiber integrated detection scheme achieved by enhancing the light response with a carbon nanotube hybrid structure [35], and revealing the key mechanism of the graphene/GaAs interface state in inducing ultra-high photoelectric gain; that is, the high-density GaAs interface state serves as the hole capture center, locating the photogenerated holes at the interface. By dynamically modulating the Fermi levels of graphene through the “Fermi level alignment” effect, significant conductance changes are induced in this high-mobility channel of graphene, ultimately achieving ultra-high photoelectric gain [36]. These studies, covering multiple dimensions such as materials, processes, physical mechanisms, and device architectures, jointly constitute a solid and diverse technical system in the field of graphene heterojunction photodetectors.

In the process of moving towards practical applications, heterojunction graphene photodetectors still face multiple challenges, such as performance, process, and integration. In terms of performance, the contradiction that high responsivity and fast response speed cannot be achieved simultaneously still exists. Although graphene/quantum dot heterojunctions can achieve wide-range performance regulation from ultra-high responsivity to fast response through structural design, the inherent trade-off between the two remains the core challenge in device optimization [12]. In terms of the process, the preparation method relying on precise transfer makes it difficult to ensure large-area uniformity and yield and has insufficient compatibility with mainstream silicon-based technologies. Meanwhile, the performance of the device is significantly affected by the dynamic adsorption and desorption of the environmental atmosphere, which leads to a severe test for its long-term operational stability [34]. At the system level, existing research mostly focuses on unit devices, while the development of large-scale array integration and corresponding readout circuits required for imaging and spectral analysis lags behind.

Future research on heterojunction graphene photodetectors will focus on the following key directions: at the physical device level, new mechanisms such as asymmetric electrodes [18] and ferroelectric coupling [13] should be actively explored to simultaneously enhance the gain and response speed. At the material and process levels, it will be necessary to develop atomic-level precise interface engineering to enhance the uniformity, the stability of the devices, and their compatibility with standard processes. Ultimately, at the application level, it is necessary to promote the development of devices towards array and system integration and develop “on-chip detection” solutions for scenarios such as optical communication and intelligent sensing, thereby achieving a leap from outstanding laboratory performance to practical industrial technology.

In summary, heterojunction graphene photodetectors have achieved significant progress but still face challenges in performance, process, and integration. To systematically summarize and compare the representative works, the key device configurations, working mechanisms, detection bands, and performance metrics are summarized in Table 1, providing a clear overview of the current status and technological trends of heterojunction-type graphene photodetectors. This analysis highlights the trade-offs among different material systems and device architectures and underscores the need for coordinated advances in interface engineering, large-area fabrication, and system integration to transition these devices from laboratory demonstrations to practical applications.

### 2.2. Light Gate Effect-Type Photodetector

The optical gate effect-type graphene photodetector modulates channel conductance by obtaining photogenerated carriers through interface traps and is regarded as one of the key paths to achieving ultra-high photoelectric gain. Graphene, with its outstanding carrier mobility and flexible band regulation characteristics, has become an ideal material platform for constructing such detectors [37]. In recent years, through integration with other material systems or the structural modification of graphene itself, much progress has been made in improving the responsivity, expanding the detection band, and balancing the response speed of this type of detector.

A major research thrust in this field concerns the integration of graphene with various material systems to enhance the photogating effect. In silicon-based integrated systems, a deep understanding of the physical mechanisms of the interface is vital. The bandgap of silicon limits its photoelectric response to infrared light with wavelengths greater than 1100 nm. By taking advantage of the Urbach tail absorption and carrier trapping effect at the SiO_2_/p-Si interface and combining it with the optical gate effect to modulate channel conductance, researchers have successfully achieved wideband ultra-high response detection from visible light to near-infrared (Figure 4) [38]. The analysis of the graphene oxide–silicon structure indicates that the graphene channel has significant P-type characteristics, and its intrinsic P-type carrier concentration is much higher than that of photogenerated carriers, dominating the photocurrent. By applying a back gate bias, not only can the gate amplification of the photocurrent be achieved, but also its attenuation can be adjusted, thereby adapting it to the requirements of different readout circuits [39]. In addition, using ionic polymers as the top gate medium can effectively regulate the interface coupling state, thereby enhancing the device responsivity to a higher level [40]. To enhance the performance of infrared detectors in the communication band, research was conducted by combining graphene with materials such as PtSi and utilizing the optical gate effect to provide high gain. As a result, the responsivity (Res) of the composite detector in the 1310 nm band was increased to 1.5 A/W, achieving an order of magnitude improvement compared to traditional PtSi detectors [41].

(1) $$\mathrm{Res}=\frac{I_{\mathrm{ph}}}{P},$$

Among them, I_ph_ is the photocurrent and P is the light power incident on the detector. Another effective solution is to use amorphous silicon/crystalline silicon Fabry–Perot optical microcavities, which achieve efficient near-infrared detection at a wavelength of 1543 nm [42]. The photogate effect graphene detector based on an InSb substrate achieved a responsivity improvement of over 100 times in the mid-infrared 4.6 μm band, demonstrating its application potential in longer band detection [43].

In addition to material combination innovation, the optimization of device structure has also been proven to be an effective means for enhancing performance. For instance, the multi-channel electrode parallel structure fabricated using nanoprocessing technology effectively amplifies the measured signal without affecting the light detection range, thereby enhancing the overall photocurrent output. This structural optimization based on micro-nano processing is an effective means of enhancing device performance [44]. Recent research has focused on resolving the inherent contradiction between high gain and fast response through the integration of heterogeneous structure design and new physical mechanisms. Introducing a tunneling mechanism assisted by the optical gate effect into two-dimensional material heterojunctions enables the device to achieve a microsecond-level response speed while maintaining high responsivity [45]. Based on nanoscale graphene nanoribbon channels with considerable bandgaps, high responsiveness, high gain, and fast response were achieved in a synergistic manner using SiO_2_/lightly doped silicon interface charge interaction. This work demonstrates that when graphene is trimmed into one-dimensional nanoribbon structures, it can introduce a direct bandgap and enhance light absorption while maintaining efficient light gate control capabilities [46]. Another strategy for enhancing mobility is using a precisely controlled nanoscale random layer stacking of graphene. This artificial microstructure maintains excellent electrical properties while effectively suppressing substrate scattering, thereby improving the optical response performance of the device [37]. In addition, theoretical research has revealed two carrier dynamics regions dominated by either a direct current field or an alternating current field by establishing a nonlinear direct current photoconductive model of graphene under strong field drive, laying an important theoretical foundation for the design of non-equilibrium quantum optoelectronic devices [47]. These studies collectively demonstrate that, from one-dimensional nanoribbon channels to precise stacking structures and then to micro-nano electrode design, nanoscale materials and structural engineering have become an indispensable means for optimizing the performance of light gate effect detectors.

**Figure 4:**
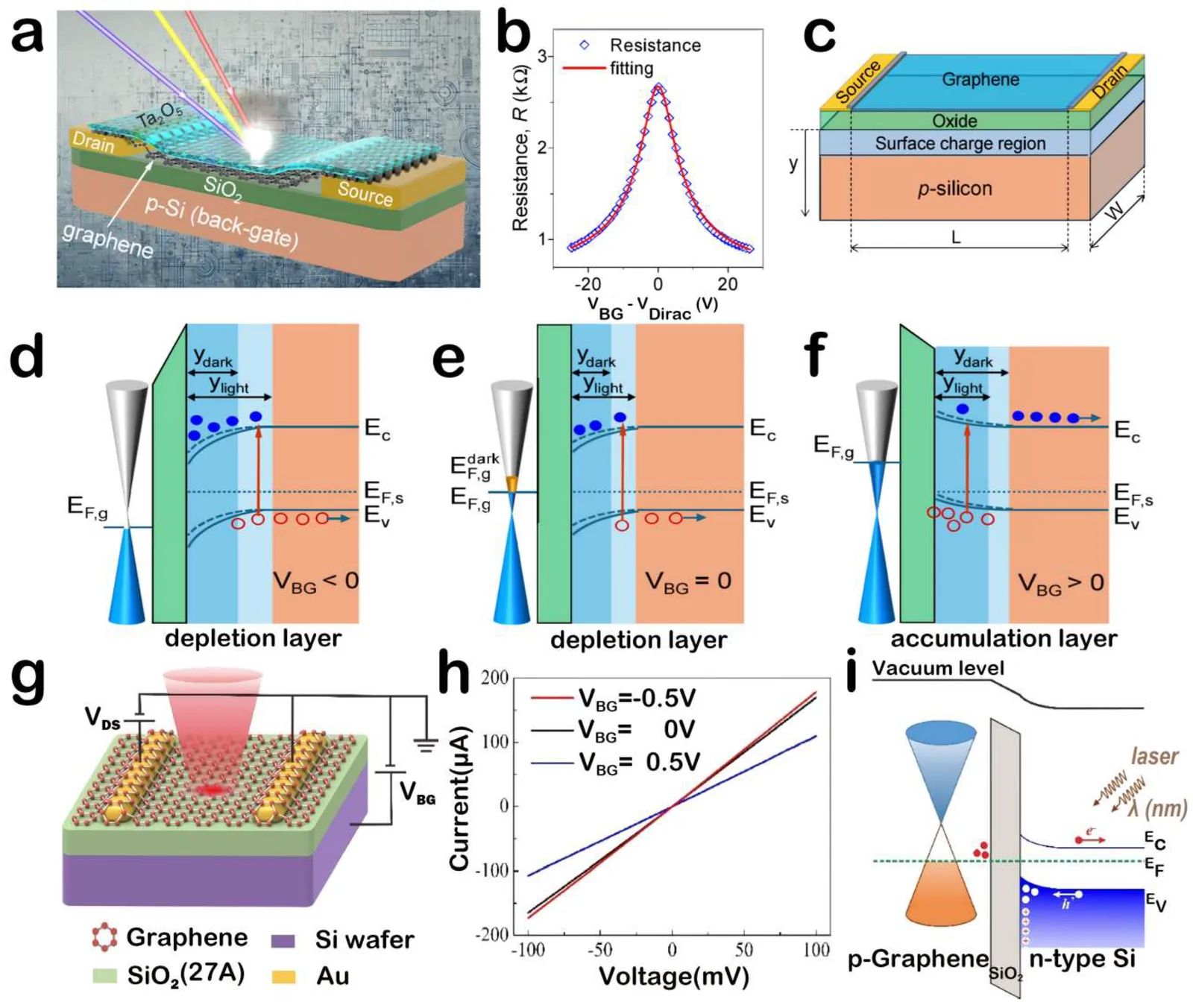
Schematic diagram of the structure and performance characterization of graphene-based photodetectors. (**a**–**f**) Photonic gated effect graphene field-effect transistor photodetector based on P-type Si substrate. (**a**) Schematic diagram of the device structure with an Al_2_O_3_/graphene/SiO_2_/p-Si stacked structure and source and leakage electrodes. (**b**) Resistance-voltage transfer curve under dark state conditions. (**c**) Schematic diagram of device structure parameters. (**d**–**f**) Band diagrams of graphene/SiO_2_/p-Si heterojunctions under different back gate voltages. Graphics copied with the permission of the reference materials [38]. All rights reserved, 2025, American Chemical Society. (**g**–**i**) Optical response characteristics of graphene-oxide-silicon structure. (**g**) Schematic diagram of the device layer structure and electrical measurement. (**h**) I–V traces graphene layers with a resistance of approximately 600 Ω. (**i**) Schematic diagram of the energy band of P-type graphene/SiO_2_/n-Si. Graphics copied with the permission of the reference materials [39]. All rights reserved 2023, Multidisciplinary Digital Publishing Institute.

Despite these remarkable achievements, this field still faces several key challenges determined by the core physical mechanisms and material properties. The contradiction between the response speed and gain is rooted in the physical nature of the optical gate effect. Achieving high photoelectric detection gain depends on a long photogenerated carrier lifetime, which fundamentally limits further improvements in the response speed [45]. High light intensity subsidence leads to center saturation, resulting in a decrease in responsivity and the failure of the linear relationship. This usually makes the linear dynamic range of the device relatively narrow, and its ultra-high responsivity is often maintained only at extremely low light intensity. As the light intensity increases, the interface subsidence center tends to saturate [41]. In addition, the long-term stability and reliability issues of performance are rather prominent. Graphene and its heterogeneous interfaces are extremely sensitive to the external environment, and the trapped state distribution is prone to drift [39]. Finally, the complex heterostructures and fine nanofabrication processes adopted to achieve high performance have insufficient compatibility with standard semiconductor processes, which hinders their large-scale integrated application [40].

To address the above challenges, future research will tend towards multi-dimensional collaborative innovation. Exploring the novel collaborative mechanism between the optical gate effect and ultrafast tunneling and other processes is the key to breaking through the response speed bottleneck [45]. The development and utilization of interface engineering strategies for the real-time control of sink energy states by gate voltage is expected to achieve a wider linear response range [39]. Efforts to enhance stability will focus on developing new stable interface layers or two-dimensional insulating materials [42]. In addition, deepening theoretical understanding and simulation will strongly guide the “on-demand design” of high-performance devices [47]. These continuous efforts aim to promote the transition of photonic gate effect graphene photodetectors from the laboratory to practical applications, enabling them to play a significant role in future high-speed optical interconnection, on-chip spectral sensing, and next-generation infrared imaging fields.

Photogating-type graphene photodetectors have seen considerable progress in responsivity, detection band, and response speed. Representative work is summarized in Table 2, grouped into Si-based, III-V and 2D material, and hybrid/engineered photogating systems. This comparison highlights the trade-offs among different material systems and device architectures and underscores the need for coordinated advances in interface engineering, material integration, and device optimization.

### 2.3. Photogating Heating/Plasma Graphene Photodetector

Photothermal-electric/plasma-enhanced graphene photodetectors are an important development direction in the current field of photodetection. The core lies in achieving carrier separation at zero bias voltage by utilizing the photothermal-electric effect and significantly enhancing the interaction between light and graphene through the nano-plasma structure, thereby making breakthroughs in response speed, operating bandwidth, and power consumption control. Such devices not only demonstrate the unique advantages of graphene as an optoelectronic material but also provide a new realization path for integrating photonics with high-performance sensing systems.

Device architectures for photothermoelectric and plasmonic enhancement are relevant. In recent years, a variety of innovative structures have been proposed to enhance the efficiency of photothermal and electrical conversion and strengthen the plasma resonance effect. For example, the parabolic energy barrier structure based on nano-perforated graphene layers has achieved efficient detection at room temperature in the terahertz band, and its response rate can be further improved to above 0.10 A/W through plasma resonance [48]. Similarly, the combination of single-layer graphene with metamaterial perfect absorbers and asymmetric T-shaped resonators achieved a peak absorption rate of over 80% in the near-infrared band. By leveraging the photothermal-electric directional current mechanism, a 3 dB bandwidth of over 400 GHz was obtained at zero bias and room temperature, demonstrating its potential as an ultra-high-speed photodetector. The four-port device structure (Figure 5a), simulated electric field response (Figure 5b), and SEM image (Figure 5c) validate the design, while the polarization-dependent current responses of the two channels at 1310 nm and 1490 nm (Figure 5d) further indicate its polarization-sensitive capability [49]. In terms of integration, the non-volatile graphene p-i-n homojunction detector based on a silicon photonic crystal waveguide achieves electrical programming of the homojunction via a ferroelectric dielectric layer. This device structure (Figure 5e) and the corresponding Seebeck coefficient gradient with hot carrier temperature distribution on the graphene layer (Figure 5f) collectively enable a high responsivity of 193 mA/W and a bandwidth of 17 GHz under zero bias, highlighting its application potential in a low-power system-on-chip [50]. These studies show that manipulating the distribution of light and thermal fields through nanoscale structural design is the key to achieving efficient photothermal-electrical conversion and ultrafast responses.

We now look at strategies for performance optimization via plasmonic engineering. To enhance the comprehensive performance of devices, the plasma enhancement mechanism and process optimization have been widely explored. After optimizing the waveguide-integrated structure using the particle swarm optimization algorithm, the graphene detector can achieve an A current responsiveness of 3.72 A/W in the 1550 nm band, and the noise equivalent power (NEP) is reduced to 4.54 pW/$\sqrt{Hz}$. These achievements demonstrate the important role of the design method in performance improvement [51].

(2) $$\mathrm{NEP}=\frac{V_{\mathrm{th}}}{R_{V}}=\sqrt{\frac{4k_{B}TR_{G}}{R_{V}}},$$

Among them, V_th_ is the root mean square value of the thermal noise voltage, R_V_ is the voltage responsivity, and R_G_ is the resistance of the graphene thin layer. In addition, the use of novel components with gold/graphene Sierbines base blanket plasma fractal nanostructures to excite multimodal plasma resonance can significantly enhance light absorption over a wide spectral range, with the responsivity increased by approximately 100 times at most [52]. In the field of infrared detection, the heterojunction structure that combines macroscopically assembled graphene nanofilms with gold-silicon plasma nanostructures, through the synergistic effect of the local light field enhanced by local surface plasmon resonance and the photothermal ion emission effect, achieves a responsivity improvement of thousands of times in specific bands, demonstrating an effective strategy for enhancing spectral selectivity [53]. This type of nanoengineering, including algorithm-optimized nanowaveguides, artificial fractal structures, and plasma nanoarrays, provides systematic solutions for directionally enhancing light–matter interactions and breaking through intrinsic absorption limitations.

Regarding applications in wide-spectrum detection and functional integration, this type of detector also demonstrates outstanding advantages in wide-spectrum detection and functional integration. The flexible graphene detector adopts an asymmetric forbidden electrode design, achieving an ultra-wideband response from ultraviolet to millimeter bands on a PET flexible substrate, and maintaining stable performance in a bent state, providing the possibility for wearable health monitoring and flexible imaging (Figure 6) [54]. Similarly, three-dimensional graphene foam, with its porous conductive network and high thermal conductivity, achieves a flat spectral response from ultraviolet to microwave bands at room temperature, with a responsivity of up to 10^3^ A/W [55]. In addition, by regulating the near-field distribution and Seebeck coefficient (S) gradient through metasurface structures, graphene detectors can simultaneously achieve an artificial photovoltaic response and polarization state analysis in the mid-infrared band, completing polarization detection without external calibration, thus expanding their applications in free-space communication and multi-dimensional sensing [56].

(3) $$S=\frac{\pi^{2}}{3}\frac{k_{B}}{e}k_{B}T\left(\frac{\partial \ln\sigma }{\partial n}\frac{\partial n}{\partial E_{F}}\right)$$

This formula indicates that the Seebeck coefficient S essentially measures the rate of change in electrical conductivity with respect to carrier concentration, where $k_{B}$ is the Boltzmann constant, $\frac{\partial \ln\sigma }{\partial n}$ represents the rate of change in electrical conductivity σ with respect to the logarithm of the carrier concentration n, and $\frac{\partial n}{\partial E_{F}}$ is the density of states.

Despite these significant advances, photothermal and plasmon-enhanced graphene photodetectors still face significant challenges in their practical applications. The inherently low light absorption rate of graphene limits further improvements in its responsivity. The ultrafast scattering process of hot carriers in graphene also makes it difficult to significantly increase the photothermal conversion efficiency [57]. In addition, the reliance on complex nanostructures to achieve high performance poses a severe test for the consistency of the fabrication process [51]. At the same time, the integration of these structures with standard CMOS processes still presents difficulties, such as the limitations on device response speed and bandwidth, and the incompatibility of device structures with CMOS design rules [52].

The future development path for addressing these challenges has become clear. More efficient optical coupling can be achieved by constructing composite structures such as graphene and photonic crystals [50]. Regulating the dynamics of thermal carriers through band engineering is a key direction for enhancing conversion efficiency [58]. The development of wafer-level transfer technology will promote the large-scale fabrication of devices [59]. Promoting the integration of detectors towards polarization and wavelength resolution can expand their application in intelligent sensing [56]. These advancements will eventually enable such devices to play a more significant role in high-speed communication and flexible wearables.

Representative photothermoelectric and plasmonic-enhanced graphene photodetectors are summarized in Table 3, covering plasmon-enhanced, waveguide-integrated, and photothermoelectric-dominant types. Key information on material systems, working mechanisms, detection bands, and performance metrics is provided for comparison.

**Figure 6:**
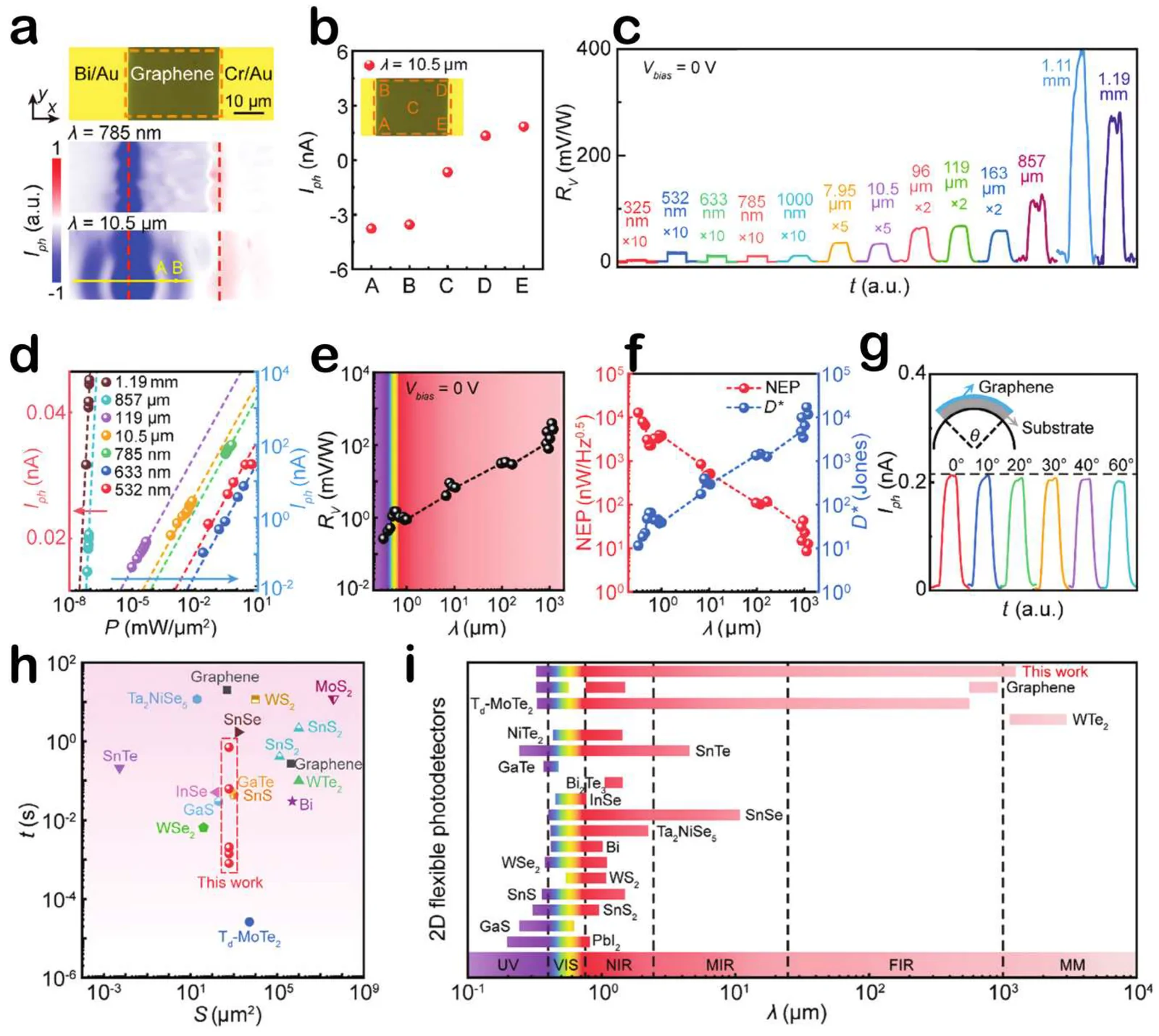
Optical detection performance of flexible graphene devices. (**a**) Optical microscope images and scanning photomicroscope images of flexible graphene photodetectors. (**b**) Photocurrent responses recorded at five typical positions within the graphene channel. (**c**) Time-resolved photocurrent response in the broadband spectral range. (**d**) Dependence of photocurrents of different wavelengths on illumination power. (**e**) Wavelength-dependent RV of flexible graphene photodetectors. (**f**) The NEP and D* of the flexible graphene photodetector are functions of the illumination wavelength. (**g**) Time-resolved photocurrent responses of photodetectors at different bending angles. (**h**,**i**) Comparison of graphene-based flexible photodetectors with other two-dimensional material flexible photodetectors in terms of response time, device area, and operating wavelength range [54]. All rights reserved 2024, Wiley-VCH Verlag GmbH & Co. KGaA.

Based on their dominant working mechanisms, graphene photodetectors can be grouped into three main types, which are schematically compared in Figure 7. The heterojunction type adopts a vertical graphene/semiconductor/metal stack, where graphene is typically synthesized by chemical vapor deposition on a metal substrate and then transferred onto a semiconductor layer such as Si, ZnO, or quantum dots, followed by metal electrode deposition. The built-in electric field at the heterointerface drives carrier separation, and graphene serves as a high-mobility transport layer. The photogating type features a lateral field-effect configuration, in which a graphene channel is integrated atop a trap-rich dielectric layer and connected to source/drain electrodes, with a back gate formed beneath the dielectric; the graphene can be obtained by CVD growth followed by transfer or by direct growth on the dielectric, while the trap layer and electrodes are defined by standard deposition and lithography processes. Trapped photogenerated carriers modulate the channel conductance via capacitive coupling, enabling high gain. The photothermoelectric/plasmonic type consists of a planar graphene sheet with asymmetric metal contacts or integrated plasmonic nanoantennas, where the graphene is synthesized by CVD or exfoliation, the asymmetric electrodes are deposited by photolithography, and the plasmonic nanoantennas are patterned by electron-beam lithography. A light-induced temperature gradient or hot-carrier effect generates a photovoltage at zero bias. A systematic comparison of their core mechanisms, key advantages, key limitations, and typical applications is provided in Table 4 at the end of Chapter 2. These examples illustrate that the synthesis of high-quality graphene and its integration with functional materials are critical to realizing each device architecture, directly influencing interface quality, contact resistance, and carrier transport, and thus form the foundation for the performance optimization discussed throughout this review.

## 3. Performance Breakthroughs and Application Frontiers of Graphene Photodetectors

Graphene photodetectors, leveraging the inherent structural and performance advantages of the material, have achieved multi-dimensional performance breakthroughs in technological innovation and application expansion. Their research and development have gradually moved from basic performance optimization to the following four core application frontier directions: adaptation to extreme environments, flexible wearability and large-scale integration, multifunctional integration and intelligent sensing, and full-band detection from ultraviolet to terahertz. It has become a key core component for breaking through the bottleneck of traditional photoelectric detection technology and supporting the development of the new generation of optoelectronic systems. In the field of extreme environment monitoring, graphene-based photodetectors, relying on their excellent thermal stability, mechanical strength, and potential for band regulation, have achieved high sensitivity, wide-spectrum responses, and stable operation under harsh conditions such as high temperature, high pressure, strong radiation, and complex corrosion through innovations in heterojunction engineering, micro-nano integration, and packaging technologies. At the same time, they are evolving towards self-adaptation and intelligent perception. They can meet the sensing demands of major strategic fields such as aerospace, deep space exploration, and energy exploration; in the directions of flexibility, wearability, and large-scale integration, the inherent mechanical flexibility of graphene can be taken advantage of. By integrating a low-dimensional material composite, low-temperature green growth and flexible substrate integration process innovation, the device has achieved a balance between high performance and high flexibility. Meanwhile, the breakthrough in wafer-level large-scale preparation technology has laid a process foundation for its application from laboratory samples to civilian and industrial practical applications such as health monitoring and portable imaging. In the field of multifunctional integration and intelligent sensing, the research paradigm of graphene photodetectors has been upgraded from single signal conversion to on-chip intelligent nodes. Through heterogeneous integration with non-volatile storage and photonic platforms, combined with all-optical modulation, metasurface assistance and other technical means, the device is endowed with capabilities such as information storage, processing, and high-dimensional optical information analysis. It provides core support for building low-power intelligent optoelectronic systems; in the full-spectrum detection direction from ultraviolet to terahertz, graphene-based devices have broken through the band limitations of traditional detectors by leveraging strategies such as heterostructure construction, band engineering, light field regulation, and waveguide integration. They have achieved comprehensive performance improvements, high responsiveness, high detection rates, and rapid responses across the entire spectral range, including deep ultraviolet, vision-near-infrared, mid-infrared, and far-infrared to terahertz. At the same time, compatibility and integration with silicon-based CMOS processes promote the development of devices towards miniaturization, chipization, and high speed, meeting the wide-spectrum detection requirements of multiple fields such as spectral analysis, optical communication, and security detection. The research and development of the four major application frontiers not only fully explores the material property advantages of graphene, but also continuously breaks through the core bottlenecks in device preparation, performance regulation, and system integration through cross-disciplinary and cross-scale technological innovation, promoting the evolution of graphene photodetectors from single-function devices to a new generation of high-performance, multifunctional, integrated, and practical optoelectronic devices. This has opened up a brand-new path for technological innovation and industrial development in the field of photoelectric detection.

### 3.1. Extreme Environment Monitoring

Extreme environment monitoring is a core technical demand in fields such as aerospace, deep space exploration, energy exploration, and advanced industrial manufacturing. Such environments are often accompanied by harsh conditions such as continuous high or low temperatures, extreme pressure, strong radiation, and complex chemical corrosion, which pose a huge challenge to the long-term stability and reliability of sensing equipment. Two-dimensional materials represented by graphene, with their atomic-level thickness and nanoscale interface effects, extremely high carrier mobility, outstanding thermal stability, excellent mechanical strength, and flexible band regulation potential, provide a revolutionary material platform for the development of a new generation of photodetectors resistant to extreme environments [60]. Through heterojunction engineering and micro-nano integration technology innovation, graphene optoelectronic devices are becoming a key technology for achieving high sensitivity, wide-spectrum responses, and intelligent perception under harsh conditions.

The intrinsic stability of graphene in high-temperature environments endows it with unique advantages in thermal sensing and photoelectric detection. The thermal resistance temperature sensor constructed based on the chemical vapor deposition of single-layer graphene exhibits a stable positive temperature coefficient and “metallic” properties within a wide temperature range of 300 to 575 K. Its high-temperature resistance temperature coefficient (TCR) is as high as 2.39 × 10^−3^ K^−1^, making it suitable for industrial monitoring scenarios exceeding 500 K, such as oil exploration and pharmaceutical sterilization [61].

(4) $$\mathrm{TCR}=\frac{1}{R_{0}}\cdot \frac{R-R_{0}}{T-T_{0}},$$

This formula is used to quantify the sensitivity of the material’s resistance to temperature changes, where R represents the resistance value at temperature T. In the field of high-temperature photodetection, a WSe_2_ field-effect transistor detector using hexagonal boron nitride fully encapsulated graphite sheet electrodes has achieved a significant breakthrough. This device can withstand a temperature of 700 °C in the air and exhibits an unconventional negative photoconductivity phenomenon at 400 °C, with a responsivity of an astonishing 2.2 × 10^6^ A/W [62]. To break through the fundamental limitation of zero bandgap in graphene, researchers innovatively adopted high-pressure-induced phase transition technology and successfully prepared a stable “diamond-like carbon” semiconductor phase with a wideband gap of approximately 2.7 eV at a high pressure of approximately 6.4 GPa. This structure performs stably within a wide temperature range of 80–420 K, laying a new material foundation for the detection of high-pressure extreme environments. This achievement highlights the crucial role of nanoscale van der Waals heterojunctions and interface constraints in realizing new stable phase-change materials [63].

In extreme environmental detection in specific bands, such as deep ultraviolet and solar-blind ultraviolet, graphene plays a crucial role as a transparent electrode or interface layer. The optimized interdigital graphene electrode/p-silicon carbide metal-semiconductor metal ultraviolet photodetector effectively reduces the electrode shadow effect by taking advantage of the high light transmittance and low reflectivity of graphene, maintaining a light-to-dark current ratio of up to 270 at a high temperature of 500 K [64]. Similarly, the solar-blind ultraviolet detector integrated with a thermally reduced graphene oxide electrode and high-alumina component AlGaN has a breakdown voltage exceeding 400 V and is suitable for complex industrial environments with strong electrical interference [65]. The detector constructed by combining graphene as the top electrode with a vertical (Al,Ga)N nanowire array effectively overcomes the carrier confinement effect of the AlN barrier layer through the horizontal carrier transport mechanism promoted by the core-shell structure [66]. In addition, the composite film detector made by dispersing graphene in a self-healing polymer matrix can withstand a tensile strain of 1000% and fully recover its performance after damage, providing a highly robust solution for field environments with mechanical damage risks. This device demonstrates that combining nanomaterials and polymers at the nanoscale can realize outstanding adaptability and self-repairing ability in extreme mechanical environments [67].

Realizing ultra-wideband photoelectric responses from ultraviolet to terahertz is an important direction for meeting the multispectral perception requirements in complex environments. Detectors based on twisted single-layer-bilayer graphene Moire superlattices exhibit significant photocurrent responses in the visible light to terahertz bands through gate voltage regulation, with noise-equivalent power as low as 27 fW/Hz^1/2^ at a low temperature of 4.5 K [68]. The Bi_2_Se_3_-PtSE_2_ van der Waals heterojunction integrates multiple physical effects, achieving ultra-wideband detection from ultraviolet to terahertz, and maintaining stable performance within an extremely wide temperature range of 183–501 K [69]. For low-cost manufacturing, the black phosphorus/graphene oxide van der Waals heterojunction prepared by the solution method still maintained a responsivity of 12 A/W at 400 K [70], while the graphene-ZnO Schottky junction constructed on a flexible substrate also demonstrated a responsivity of 0.49 A/W and good flexibility [71]. The technical boundaries of sensing in extreme environments has been jointly expanded by other new material systems, such as SNSE-based detectors, with their sensitive responses to environmental molecules, and ultraviolet light detectors based on polymer microarrays for wearable medical devices (Figure 8) [72], as well as the excellent corrosion resistance of ZR-doped amorphous Ga_2_O_3_ flexible detectors, with their excellent corrosion resistance [73].

Extreme environment monitoring is developing towards intelligence and self-adaptation. Based on the high-temperature-resistant sun-blind ultraviolet photodetector, a responsivity of up to 67.5 A/W can still be achieved in a high-temperature environment of 610 K, and it has the ability of optical signal encryption processing, providing a feasible hardware basis for edge intelligent perception systems facing high-temperature extreme environments [74]. The VO_2_/MoTe_2_ heterojunction detector integrates phase-change materials with two-dimensional materials to achieve adaptive switching among three working modes—a p-n junction, Schottky junction, and radiometer in a single device—covering wide-spectrum detection from visible light to medium and long-wave infrared [75]. These innovations indicate that photodetectors for extreme environments are evolving from single-signal conversion units to intelligent perception systems with initial information processing and decision-making capabilities.

The current research and development of graphene-based photodetectors for extreme environments mainly faces three major challenges. First, there is a prominent contradiction between performance, tolerance, and the preparation process. For instance, the method of opening the bandgap through high-pressure-induced phase transition is subject to harsh conditions and is difficult to implement practically [73]. However, the long-term environmental stability of heterojunctions prepared by simple processes such as solution methods is confronted with deep-seated issues, including how to suppress the intrinsic degradation of BP materials at room temperature, how to ensure the long-term pollution-free stability of the liquid-phase process interface, and how to achieve effective packaging without undermining the advantages of simple processes. These tests of “long-term environmental stability” are precisely the key bottlenecks that restrict the transition of such high-performance detectors from the laboratory to engineering applications [70].

Second, the adaptability of the device to the complex extreme environment of multi-physics field coupling is seriously insufficient. Most existing studies focus on a single stress factor and lack the simulation and verification of real and complex environments, such as concurrent high temperature and high pressures. A reliability assessment system has not yet been established [75]. Finally, intrinsic noise suppression under extreme conditions has become a technical bottleneck. High temperatures cause a sharp increase in dark current [64], while the detection in terahertz and other bands is severely interfered with by background noise [68], posing a serious challenge to the improvements in the signal-to-noise ratio.

**Figure 8:**
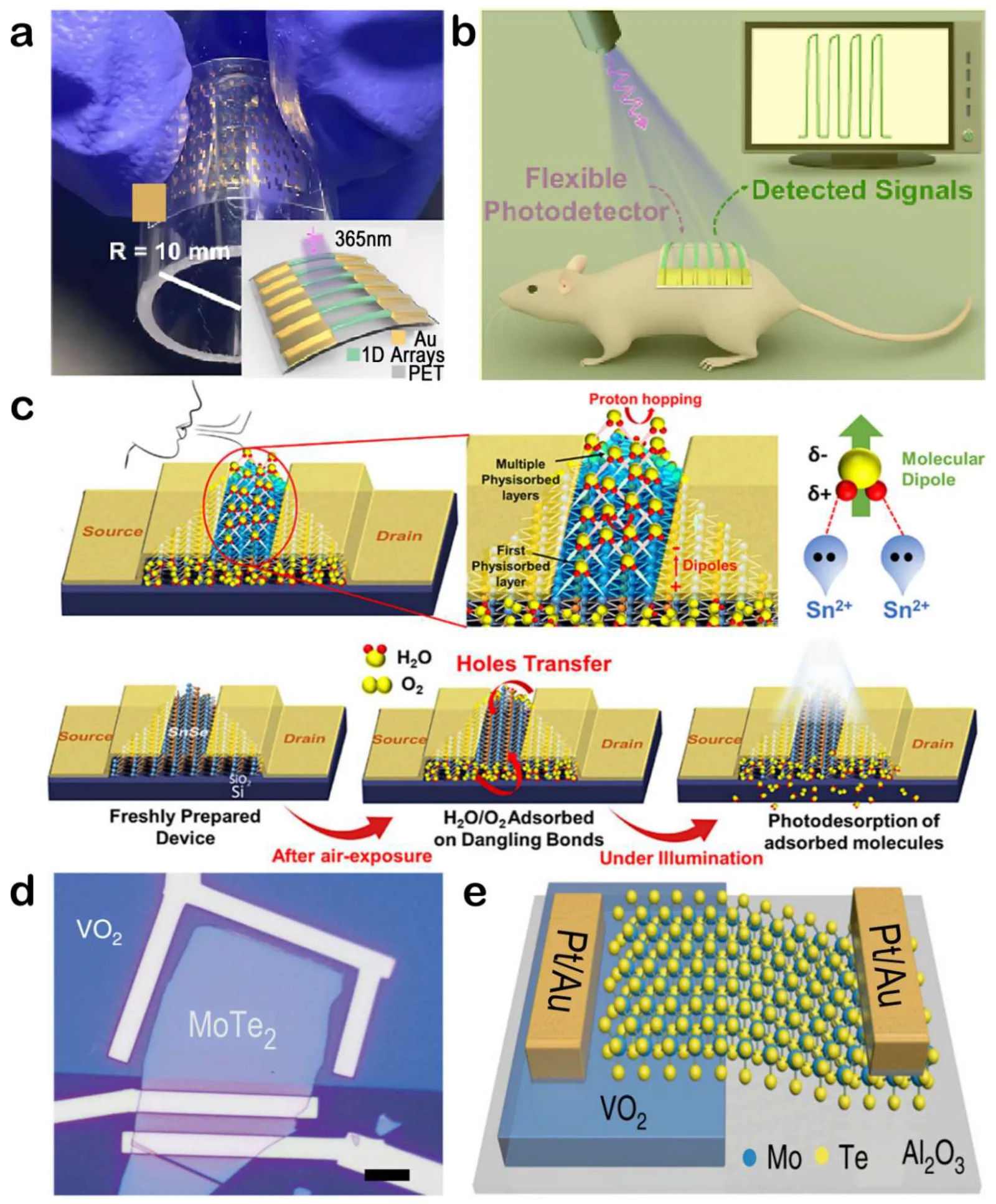
Working mechanism and structural characterization of photodetectors with different structures. (**a**) Schematic diagram of the structure of the ultraviolet detector based on polymer microline array. (**b**) Schematic diagram of a flexible photodetector monitoring ultraviolet detection signals. (**c**) Schematic diagram of the structure of the Sb_2_O_3_/PdTe_2_/Si heterojunction photodetector and its self-powered electro-optic response mechanism. Graphics copied with the permission of the reference materials [72]. All rights reserved, 2024, MDPI AG. (**d**,**e**) Schematic and optical micrograph of the VO_2_/MoTe_2_ heterostructure on a sapphire substrate. Scale bar, 10 μm. Graphics copied with the permission of the reference materials [75]. All rights reserved, 2020, Springer Nature.

The future development of technology to address the above-mentioned challenges presents a clear path. At the nanoscale, in situ performance regulation through means such as programmable heterojunctions, stress engineering, and molecular doping will become the key to breaking through the contradiction between process and tolerance. To address the challenge of balancing performance, process, and tolerance, research will shift to the development of novel programmable heterojunctions, achieving in situ performance regulation through mild means such as stress engineering and molecular doping, to avoid extreme preparation conditions [65]. To address the adaptability issue in complex extreme environments, it is necessary to establish a material failure theory under multi-field coupling and use in situ characterization and simulation to study interface behavior, providing guidance for designing inherently robust device architectures [69]. To address the bottleneck of extreme noise suppression, the integration of sensing and computing and edge intelligence have become key. It is necessary to develop intelligent detectors with front-end signal processing capabilities to compress data and suppress noise [74]. Meanwhile, the development of fully encapsulated technology based on high-quality two-dimensional insulating materials will enhance the long-term operational reliability of the system in real harsh environments from a physical perspective [62]. Through interdisciplinary collaborative innovation, this field is expected to provide a reliable technical foundation for the country’s major strategic demands.

Representative applications of graphene photodetectors in extreme environment monitoring are summarized in Table 5, covering high-temperature, high-pressure/mechanical damage, UV/solar-blind, and corrosive/complex environments. The table lists the application scenario, core contribution, and key technology for each case, highlighting recent progress in this field.

### 3.2. Flexibility, Wearability and Large-Scale Integration

Flexibility, wearability, and large-scale integration are the key directions for promoting the transition of graphene photodetectors from the laboratory to practical applications. Graphene, with its inherent mechanical flexibility, high carrier mobility, and excellent chemical stability, has become the ideal core material for constructing the next generation of flexible optoelectronic devices. In recent years, researchers have made important progress in achieving high performance, high flexibility, and the large-scale fabrication of devices by compounding graphene with zero-dimensional quantum dots, other two-dimensional materials, and other nano-functional components, and developing advanced integration processes for flexible substrates, laying a solid foundation for applications in fields such as health monitoring, portable imaging, and industrial inspection.

Existing research has confirmed the diversity and performance potential of graphene photodetectors on flexible substrates. In the field of flexible wearable applications, ultra-wideband detectors operating at zero bias voltage can be constructed by integrating single-layer graphene and asymmetric metal electrodes on PET flexible substrates, with a response range extending from the ultraviolet to millimeter-wave bands [54]. By using polyimide as a flexible substrate and in situ growing graphene with PbS quantum dots to form heterojunctions, high-responsivity detectors that maintain stable performance after repeated bending can be fabricated [76]. To achieve the comfort and self-powering capability of wearable devices, researchers have constructed a semi-transparent self-powering photodetector with both high light transmittance and good flexibility by spin-coating reduced graphene oxide films on PET substrates [77]. In terms of large-scale integration, researchers have successfully integrated wafer-level graphene as an insertion layer with germanium substrates to fabricate high-performance Schottky junction photodetectors, providing a feasible path for achieving large-scale production [78]. Furthermore, a full-process technology on a 200 mm wafer platform was established for the large-scale production of graphene–quantum dot hybrid photodetectors. This process not only demonstrates high yield but also achieves high integration, successfully fabricating imaging sensor array chips and completing sealed packaging, providing a feasible path for system integration [79].

The current innovation in development is mainly reflected in the low-temperature and green growth of graphene materials as well as the novel design of device structures. To achieve low-temperature compatibility with flexible electronics processes, researchers have developed a PECVD technology that uses benzene as the carbon source to synthesize high-quality graphene on copper foil pre-treated with CO_2_ laser to reduce the surface roughness at 400 °C through optimized growth and annealing processes [2]. Another innovation is to directly grow graphene on sapphire substrates by plasma-assisted chemical meteorological deposition and further integrate it with MoS_2_ to construct heterojunction detectors [80]. Environmentally friendly and low-cost preparation strategies are also highly valued, such as the synthesis of transfer-free eco-friendly graphene using natural camphor as a carbon source and its integration with CsPbBr_3_ perovskite quantum dots. The hybrid structure (Figure 9a), the energy band alignment at the interface (Figure 9b), and the device geometry (Figure 9c) collectively facilitate efficient charge separation and photoresponse [81]. In terms of device structure design, laser-induced graphene technology can be used to directly pattern porous electrodes on polyimide, providing a platform for the construction of customizable flexible heterojunction devices. To enhance the response, the QD-LIG heterostructure schematic before/after ligand exchange (Figure 9d), photo-to-dark current ratio versus deposition cycles (Figure 9e), I–V curves under dark/light (Figure 9f), responsivity and external quantum efficiency (EQE) versus irradiance at 10 V (Figure 9g), and detectivity versus irradiance (Figure 9h) are systematically examined [82]. To enhance the response performance of the device, graphene nanowalls are adopted as photosensitive materials. Its unique vertical nanosheet structure forms numerous inner walls, where light undergoes multiple reflections, significantly enhancing the absorption and response to infrared light. This work indicates that constructing three-dimensional nanostructures is an effective nanoengineering strategy for breaking through the in-plane light absorption limit of two-dimensional materials and achieving efficient light capture [83]. For the graphene oxide system, green reduction with vitamin C effectively removes the oxygen-containing functional groups on the surface of graphene oxide and repairs the sp^2^ carbon network in its conjugated structure, thereby significantly improving the material’s electrical conductivity and photogenerated carrier mobility and ultimately greatly enhancing the light response performance [84]. Meanwhile, the introduction of PVA polymer for modification can optimize film morphology. This further achieves high-performance detection under low power consumption [85]. In summary, from the low-temperature growth of high-quality graphene films to the composites with various low-dimensional nanomaterials, and then to nanofabrization technologies such as laser-induced processing, these material and process innovations at the nanoscale jointly constitute the cornerstone for the performance breakthroughs of flexible graphene optoelectronic devices.

The core challenges faced by this field in moving towards mature applications are multi-level and interrelated. In terms of technical challenges, the primary issue is to resolve the contradiction between performance uniformity and high yield in large-scale production [79]. Secondly, it is necessary to enhance the long-term reliability of the device in real wearable environments, including its tolerance to mechanical fatigue, environmental erosion, and temperature changes [86]. The technological challenge lies in how to perfectly combine the preparation of high-performance materials with low-cost, high-throughput manufacturing technologies [81]. The application challenges are even more prominent. Currently, there is a lack of standardized test protocols and large-scale application scenario verification data for flexible photodetectors, including the absence of unified standards in all aspects, from optical power calibration and effective area definition to noise measurement, which makes the technological transformation path unclear [54].

Looking ahead, flexible, wearable, and large-area integrated graphene photodetectors have broad prospects for development. With the continuous advancement of materials science and micro-nano processing technology, devices that combine high performance, high flexibility, low power consumption, and low cost are expected to be mass-produced. They can not only promote the innovation of human–computer interaction and health monitoring in the consumer electronics field [77], but also play an important role in professional fields such as industrial automation, security monitoring, and environmental perception [79]. Ultimately, the transformation from laboratory samples to market products requires in-depth collaboration among interdisciplinary teams to jointly overcome the full-chain challenges from basic materials and integrated processes to system applications, thereby truly unleashing the huge potential of graphene in the field of flexible optoelectronics.

Table 6 summarizes representative graphene photodetectors for flexible/wearable applications and large-scale integration, which are grouped into flexible wearable detection, green/low-temperature preparation, and wafer-level large-scale integration. For each category, the application direction, core contribution, and key technology are listed, providing a clear overview of recent advances in this field. These examples demonstrate the potential of graphene photodetectors for practical deployment in flexible and wearable systems.

### 3.3. Multifunctional Integration and Intelligent Sensing

In response to the demands of next-generation intelligent optoelectronic systems, the research paradigm of graphene photodetectors is shifting from performance optimization to multifunctional integration and intelligent perception. This transformation deeply exploits wide-spectrum absorption, high-speed response, and excellent heterogeneous integration compatibility of graphene, aiming to upgrade the detector into an on-chip intelligent node with information storage, processing, and communication capabilities.

In the field of multifunctional integration, combining optical detection with on-chip non-volatile memory is the key to achieving integrated memory and computing. The graphene p-i-n homojunction detector with an integrated ferrodielectric achieves zero-bias operation and long-term charge retention by utilizing the residual polarization field, providing a foundation for photoelectronic synapses [50]. The critical coupling between the microring resonator and graphene can synergistically optimize the performance of the device. Based on the structure of integrating a dual-core waveguide into a silicon microring resonator, photoelectric detection with high voltage responsivity and low noise equivalent power was achieved at zero bias voltage by taking advantage of the resonance field enhancement effect [87]. The dual-mode photodetector further expands the application flexibility. The graphene/InP van der Waals heterojunction can be electrically switched between self-powered photovoltaic mode and bias photoconductive mode through electrode design, taking into account the requirements of low power consumption and high gain [88].

Heterogeneous integration with high-performance photonic platforms is another important direction for enhancing system functionality. A graphene detector was monolithically integrated on a thin-film lithium niobate platform, successfully combining high-speed detection with the platform’s inherent excellent electro-optical modulation capability [89]. Using graphene as a transparent conductive electrode can also effectively enhance the performance of traditional heterojunctions. The introduction of graphene transparent top electrodes in NiO/Si heterojunctions significantly enhanced the carrier collection efficiency and responsivity [90]. The heterojunction composed of one-dimensional nanomaterials with controllable bandgaps, such as graphene nanoribbons and InGaZnO (IGZO), has achieved wide-spectrum self-powered detection from ultraviolet to near-infrared [91].

The core of achieving intelligent perception lies in endowing the detector with front-end information processing capabilities or programmable features. Detectors with all-optical modulation capabilities exhibit neuro-like plasticity. The photoconductive response of devices based on the CdS/graphene/Ge sandwich structure can be continuously regulated by another beam of light, laying the foundation for optical domain simulation calculations [92]. Deep integration with artificial micro-nanophotonic structures enables the detector to directly analyze high-dimensional optical information. Metasurface-assisted graphene detectors based on nanoantenna arrays can simultaneously decouple the wavelength and polarization state of incident light, achieving feature extraction at the hardware level (Figure 10) [93].

However, the development of this field still faces a series of challenges. Multifunctional integration and complex nanostructures significantly increase process complexity and manufacturing costs, which pose extremely high requirements for process consistency [50]. There are inherent trade-offs among different performance indicators. For instance, high responsiveness and high speed are often difficult to achieve simultaneously [88]. The quantum efficiency and stability of devices based on new mechanisms of nanoscale physics still need to be improved [94]. In addition, achieving high-performance and high-yield heterogeneous integration of graphene with different material platforms remains a key bottleneck restricting its large-scale application [89].

Future research needs to address these challenges through cross-scale innovation. At the process integration level, it is crucial to develop standardized and CMOS-compatible two-dimensional material integration processes [95]. At the device level, it is necessary to explore new mechanisms, such as photothermal-electric effects [96] and plasma enhancement [94], to break through the traditional performance limits. At the system architecture level, promoting the integration of new paradigms, such as programmable detectors and in-memory computing, is the direction for building low-power intelligent perception systems [92]. Through collaborative design from materials and devices to algorithms, graphene photodetectors are expected to become the core intelligent units in future optical information systems.

Representative graphene photodetectors for multifunctional integration and intelligent sensing are summarized in Table 7, covering waveguide-integrated, plasmonic-/metasurface-integrated, and heterogeneous integration types. The table lists the application direction, core contribution, and key technology for each case, highlighting recent progress toward on-chip intelligent optoelectronic systems.

### 3.4. Full-Band Detection from Ultraviolet to Terahertz

Graphene, with its unique band structure, high carrier mobility, and broad-spectrum absorption characteristics, has become an ideal material for achieving full-band photoelectric detection from ultraviolet to terahertz. In recent years, through strategies such as heterostructure construction, band engineering, light field regulation, and process integration, graphene photodetectors have made significant progress in a wide spectral range covering deep ultraviolet, visible, near-infrared, mid-infrared, far-infrared, and even terahertz, demonstrating comprehensive performance advantages such as high responsiveness, high detection rate, fast response, and band tunability. This has laid a solid foundation for the development of the next generation of high-performance and multifunctional photoelectric detection systems. In this section, the graphene-thin-film semiconductor heterojunctions (such as Si, Ge, GaN, and InSb) are discussed. In these structures, graphene mainly serves as a transparent electrode or a carrier transport layer, while the light absorption is dominated by the bulk semiconductor.

In the ultraviolet to deep ultraviolet band, the heterojunction design of graphene and wide bandgap semiconductors effectively enhance the detection sensitivity and spectral selectivity of the device. For example, the p–i–n structure composed of Gr/i-GaN/n-GaN has a responsivity of 20.6 A/W under 340 nm ultraviolet light and an extremely low dark current [97]. The hybrid-dimensional heterojunction constructed with zero-dimensional graphene quantum dots and β-Ga_2_O_3_ has a responsivity as high as 2.4 × 10^5^ A/W under 250 nm deep ultraviolet light, and the external quantum efficiency exceeds 10^8^%. This work indicates that the introduction of nanoscale quantum dots can effectively regulate the interfacial charge transfer path, becoming the key to enhancing light absorption and carrier multiplication in the deep ultraviolet band [98]. In addition, graphene, as a transparent electrode, combined with the vertical structure of MgZnO/GaN, achieved an EQE of 71.1% in the 255 nm solar-blind ultraviolet band [99].

(5) $$\mathrm{EQE}=\frac{I_{\mathrm{photo}}/e}{P_{\mathrm{inc}}/(h\nu )}=R\cdot \frac{\mathrm{hc}}{e\lambda },$$

Among them, $I_{\mathrm{photo}}$ represents the photocurrent, and $P_{\mathrm{inc}}$ represents the incident light power. In the shorter vacuum ultraviolet wavelength range, the detector based on the graphene-on-silicon structure achieves full depletion operation through an ultrathin HfO_2_ dielectric layer, with an external quantum efficiency approaching or exceeding 100% in the 120–200 nm range, and is suitable for vacuum ultraviolet and soft X-ray detection [100]. These studies indicate that engineering the interfaces between graphene and wide-bandgap materials can significantly enhance ultraviolet light absorption and carrier separation efficiency, providing high-performance device solutions for deep space exploration, environmental monitoring, and biomedical sensing.

In the visible to near-infrared band, graphene achieves an effective combination of high responsiveness and wide-spectrum detection by constructing heterojunctions with other photosensitive materials. The responsivity of the graphene-PBS heterojunction detector is higher than 1 A/W in the range of 265 nm to 2200 nm and reaches 72 A/W at 792 nm [101]. The graphene/thin silicon/graphene structure has achieved wide-spectrum detection from X-rays to near-infrared, with A responsivity of 0.56 A/W at 375 nm [102]. The graphene-covered Schottky junction achieved a high detection rate (D*) of 7.2 × 10^10^ cm·Hz^1/2^·W under 1319 nm near-infrared light through plasma enhancement of textured silicon and localized surface plasmon resonance gold nanoparticles. This strategy ingeniously combines micron-scale optical traps with nanoscale plasma units. It greatly enhances the interaction between the near-infrared light field and matter at the nanoscale [103].

(6) $$D^{*}=\frac{R}{(2q\cdot J_{\mathrm{dark}}{)}^{1/2}}$$

This formula is used to measure the detector’s ability to detect weak signals. Here, R represents the current response rate and $J_{\mathrm{dark}}$ represents the dark current density. Additionally, the graphene/GaAs heterojunction exhibits a response rate of 122 mA/W under zero bias voltage and 808 nm illumination, demonstrating the potential for self-powered near-infrared detection [104]. These devices fully utilize the high mobility of graphene and the built-in electric field within the interface, achieving the efficient separation and collection of photogenerated carriers. They have significant application value in imaging, optical communication, and night vision fields.

In the mid-infrared band, the photothermal electric effect of graphene and the integrated design of waveguides have become the key to improving detection performance. The waveguide-integrated photothermal-electric graphene detector based on the germanium silicon platform has a responsivity of 1.97 V/W at a wavelength of 3.7 μm and zero bias voltage, and the noise equivalent power is 2.8 nW/$\sqrt{Hz}$ [105]. The integrated sulfur-based glass waveguide scheme further extends the operating wavelength to 5.2 μm, achieving the equivalent power of limiting Johnson noise and GHz bandwidth [106]. The graphene/two-dimensional medium slit structure photodetector constructed by copotential engineering utilized a configurable two-dimensional potential well to enhance the optical gating effect, achieving a high responsivity of 0.2–38 A/W in the wide infrared band of 1.55–11 μm, and demonstrated polarization-sensitive detection functionality. The comparison of different broadband detection mechanisms for graphene-based photodetectors (Figure 11a), the schematic of the configurable all-dielectric metasurface graphene photodetector structure (Figure 11b), the interface potential diagrams for different dielectric surface configurations (Figure 11c), and the simulated electric field distribution under gate voltage modulation (Figure 11d) collectively elucidate the working principle of this asymmetric slit design [107]. Simulation studies also show that the Schottky heterojunction composed of graphene and HgCdTe has a responsivity of 2.60 A/W at 5.08 μm and a detectivity of 1.45 × 10^12^ cm·Hz^1/2^·W [108]. These advancements highlight the significant role of light field localization, band regulation, and waveguide coupling in high-performance mid-infrared detection, providing chip-based solutions for gas sensing, thermal imaging, and spectral analysis. In conclusion, whether it is the configurable potential well constructed by dielectric nanoslits or the subwavelength light field confinement achieved through waveguide integration, these nanoscale engineering methods have all become the key to unlocking the efficient photoelectric conversion capability of graphene in the mid-infrared band.

In the far-infrared to terahertz band, graphene, with its bandgap-free property and high carrier mobility, has become an ideal material for achieving high-performance detection at room temperature. Through antenna impedance matching and photothermoelectric effects, a terahertz detector based on quasi-free-standing bilayer graphene achieved a voltage response rate of 35 V/W and a noise equivalent power of less than 300 pW/$\sqrt{Hz}$ at room temperature [109]. Theoretical research indicates that interband photodetectors based on graphene and black phosphorus/black arsenic barrier layers can operate in the far-infrared band with wavelengths λ ≥ 6–12 μm, and the response can be extended to the terahertz range by increasing the bias voltage, with a predicted responsivity of 3.68–10.08 A/W [110]. In addition, the near-infrared detector based on the graphene/FeCl_3_ intercalated graphene heterostructure achieved a responsivity of 130 mA/W at 1700 nm, demonstrating the potential of the all-graphene structure in long-wave detection. Through structural design and effect innovation, these studies have promoted the development of far-infrared and terahertz detectors towards room temperature, high sensitivity, and wide frequency band and have broad prospects in fields such as security detection, astronomical observation, and high-speed communication.

In terms of device processing and integration, the compatibility of graphene with silicon-based CMOS technology makes its large-scale application possible. The graphene/silicon heterojunction near-infrared detector fabricated using a standard silicon process combined with graphene transfer technology shows a response speed enhanced to the nanosecond level through “isolation trap” and defect engineering and supports high-speed operation at MHz [111]. The integration of twisted bilayer graphene with SOI waveguides achieves an A responsivity of 0.65 A/W and a 3 dB bandwidth of over 65 GHz at a communication wavelength of 1550 nm, supporting data transmission of 50 Gbit/s. The simulated electric field distribution of the device (Figure 11e) and the schematic of the Si waveguide structure with input light coupling (Figure 11f) illustrate the underlying mechanism of this high-performance integration [112]. The thermal decomposition and hydrogen intercalation processes of silicon carbide can be used to prepare quasi-free-standing bilayer graphene integrated with planar antennas to achieve terahertz detection [109]. These integrated solutions not only enhance the performance of the devices but also drive the evolution of graphene photodetectors towards miniaturization, low power consumption, and high speed, laying the foundation for on-chip optoelectronic systems and optical interconnection applications.

**Figure 11:**
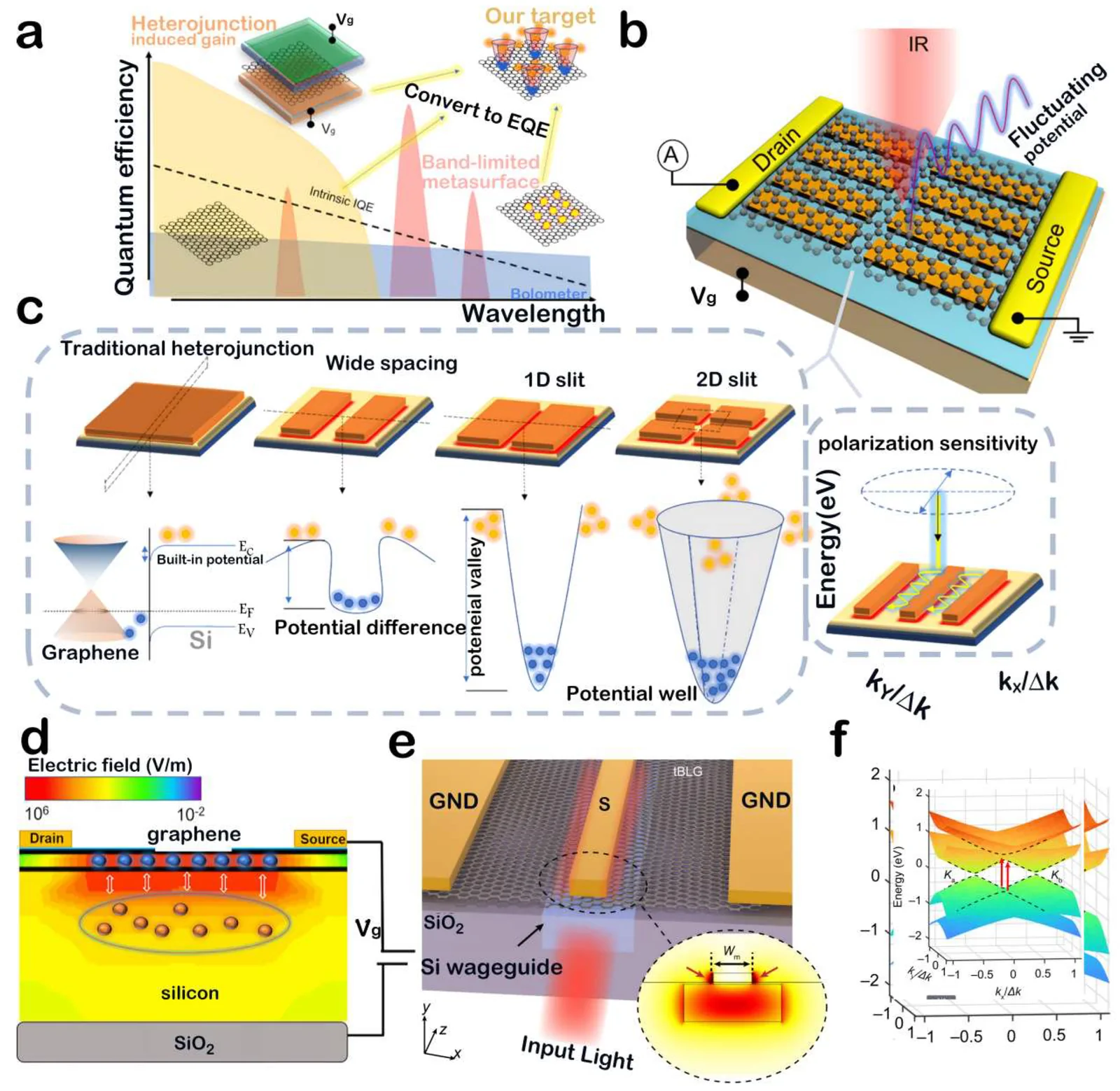
Design principle and structural characterization of graphene-based high-gain and high-speed photodetectors. (**a**–**c**) Graphene near-infrared photodetector based on copotential engineering. (**a**) Comparison of different mechanisms for broad-spectrum detection of graphene. (**b**) Schematic diagram of the structure of the configurable all-dielectric surface graphene photodetector. (**c**) Schematic diagram of the interface potential of different dielectric surface structures. (**d**) Schematic diagram of the simulation of the gate voltage on the electric field distribution. Graphics copied with the permission of the reference materials [107]. All rights reserved 2024, Springer Nature. (**e**,**f**) Waveguide integrated twisted bilayer graphene photodetector. (**e**) Schematic diagram of the structure of the twisted bilayer graphene photodetector. (**f**) Calculation of the low-band structure of bilayer graphene at a torsion Angle of 4.1°. Graphics copied with the permission of the reference materials [112]. All rights reserved 2024, Springer Nature.

Although significant progress has been made in graphene full-band photodetectors, they still face the challenges of dark current and noise suppression, especially for near-infrared and mid-infrared devices operating at room temperature, whose dark current levels still need to be further reduced [111]. The uniformity and long-term stability of device performance are also key issues. Defects introduced during the transfer of large-area graphene can affect the uniformity of the response. In terms of process integration, the large-scale combination of graphene- and silicon-based processes still faces challenges such as transfer efficiency and interface quality control—for instance, plasma damage during the graphing process, and interface state and carrier mobility degradation introduced by gate medium deposition [109]. In addition, for the far-infrared and terahertz bands, there is still room for improvement in the responsiveness and detection rate of the existing detectors.

Looking to the future, energy band engineering and interface design are expected to systematically address the above-mentioned challenges. Optimizing the passivation of the heterojunction interface is an effective technical approach for dark current suppression [99]. To enhance device uniformity, developing wafer-level van der Waals transfer technology is the key to achieving high-quality integration [106]. At the process integration level, developing a full-process CMOS-compatible manufacturing solution is the core to achieving arrayed and chip-based solutions [111]. In terms of performance improvement, the introduction of nanophotonic structures, such as plasma polaritons, can effectively enhance light absorption and improve the external quantum efficiency in specific bands [105]. For the far-infrared and terahertz bands, exploring new mechanisms based on plasma polariton resonance combined with metamaterial antenna design is a cutting-edge direction for achieving high-sensitivity detection at room temperature. Generally, with the continuous integration of multidisciplinary technologies, graphene-based full-band photodetectors will demonstrate broader application prospects in fields such as spectral analysis, high-speed communication, and quantum information.

Table 8 summarizes the full-band detection capabilities of graphene photodetectors, which are grouped into UV–deep UV, visible–NIR, and FIR–THz detection. For each category, the material/structure system, working mechanism, detection band, and key performance metrics are listed, providing a clear overview of recent advances in full-band photodetection. These examples demonstrate the potential of graphene photodetectors for wide-spectrum applications ranging from ultraviolet to terahertz.

## 4. Conclusions and Future Prospects

Graphene, with its unique two-dimensional atomic structure, zero-bandgap band characteristics, extremely high carrier mobility, and broad-spectrum light absorption capacity, provides a highly promising material platform for breaking through the performance bottlenecks of traditional semiconductor photodetectors. This article systematically reviews recent research progress in the field of graphene photodetectors and discusses it along the following two main lines: the types of core devices and performance breakthroughs. Research shows that, by constructing three major types of core devices—namely, heterojunction, optical gate effect, and photothermal-electric/plasma devices—and supplemented by nanoengineering and structural design, graphene detectors have achieved significant breakthroughs in key indicators, such as responsivity, detection band, response speed, and flexibility. The application scope has expanded from traditional wide-spectrum detection to cutting-edge fields such as flexible wearables, extreme environment monitoring, and intelligent sensing.

Despite the fruitful achievements, this study reveals that the field still faces a series of profound challenges as it moves from the laboratory to industrial application. At the physical mechanism level, the core contradiction of the current research lies in the inherent trade-off between high gain and fast response. Taking the optical gating effect as an example, the long carrier lifetime is a prerequisite for achieving high photoelectric gain, but it also fundamentally limits further improvements in the response speed. Moreover, fundamental physical issues such as non-equilibrium carrier dynamics and multi-physical field coupling have not yet been fully mastered, which restricts the precise control and optimization of device performance.

At the material and process level, large-area uniformity, process compatibility, and long-term stability are the key bottlenecks restricting industrialization. Currently, most high-performance heterojunction devices still rely on the wet transfer process of graphene. It is difficult to ensure uniformity over a large area and high yield with this process, and the residual polymers introduced during the transfer and interface contamination will seriously affect the consistency and reliability of the devices. At the same time, the mainstream fabrication processes (such as high-temperature CVD) have insufficient compatibility with CMOS back-end processes, which hinders the on-chip integration of graphene photodetectors and silicon-based readout circuits. Moreover, graphene and its heterojunctions are extremely sensitive to external environments (such as atmosphere adsorption and humidity changes), which poses a severe challenge to the long-term operational stability of the devices.

At the system application level, current research mainly focuses on the performance optimization of individual devices, while the development of array integration and accompanying readout circuits lags behind. There is a lack of a standardized device testing and evaluation system, resulting in a lack of a unified benchmark for performance comparisons among different studies. During the transition from laboratory devices to engineering systems, there are still multiple challenges such as reliability verification, packaging technology, and cost control, which hinder the practical application of graphene photodetectors in scenarios such as imaging and spectral analysis.

This study clarifies the complete technical chain from material innovation to device physics and then to system integration through the systematic integration of existing achievements. Its innovative contribution lies in the construction of a clear technical map covering core physical mechanisms and cutting-edge application scenarios. From the perspective of the integration of nanoengineering and system integration, it analyzed the core contradictions in the current research’s collaborative evolution of “performance-process-application” and pointed out the key links that need to be focused on and broken through in subsequent research, such as interface control, cross-scale manufacturing, and heterogeneous integration.

Looking ahead, the continued advancement of graphene photodetectors calls for a phased approach that prioritizes research efforts according to technical maturity and application readiness. We propose the following three-stage technology roadmap.

In the short term, efforts should be directed toward overcoming the manufacturing bottlenecks for flexible and wearable devices through wafer-scale, low-temperature growth techniques and transfer-free fabrication methods. Priority should be given to improving large-area uniformity, mechanical stability, and device yield, while establishing standardized testing protocols to facilitate fair performance comparison across different studies.

In the medium term, research should aim at resolving the inherent gain-speed contradiction and achieving large-area, uniform photodetector arrays with CMOS-compatible processes. This requires synergistic advances in photogating-assisted tunneling mechanisms, plasmonic nanostructure optimization, and waveguide-integrated device architectures for high-speed communication applications.

In the long term, the ultimate goal is to upgrade graphene photodetectors to on-chip intelligent nodes with integrated sensing, memory, and computing capabilities. Achieving this vision demands breakthroughs in heterogeneous integration with non-volatile memory materials and photonic platforms, as well as the development of programmable detectors with in-sensor processing functionality.

In summary, this three-stage roadmap outlines a coherent path toward practical graphene photodetectors. The short-term phase lays the groundwork for commercialization by addressing manufacturing scalability and device reliability, while the medium-term phase tackles the fundamental gain-speed trade-off that has long constrained device performance. The long-term vision, though ambitious, charts a direction toward intelligent optoelectronic systems where detection, memory, and computation converge on a single chip. We hope that framework provides not only a guide for future research but also a basis for dialog between academia and industry on the practical translation of graphene photodetectors.

## Figures and Tables

**Figure 1:**
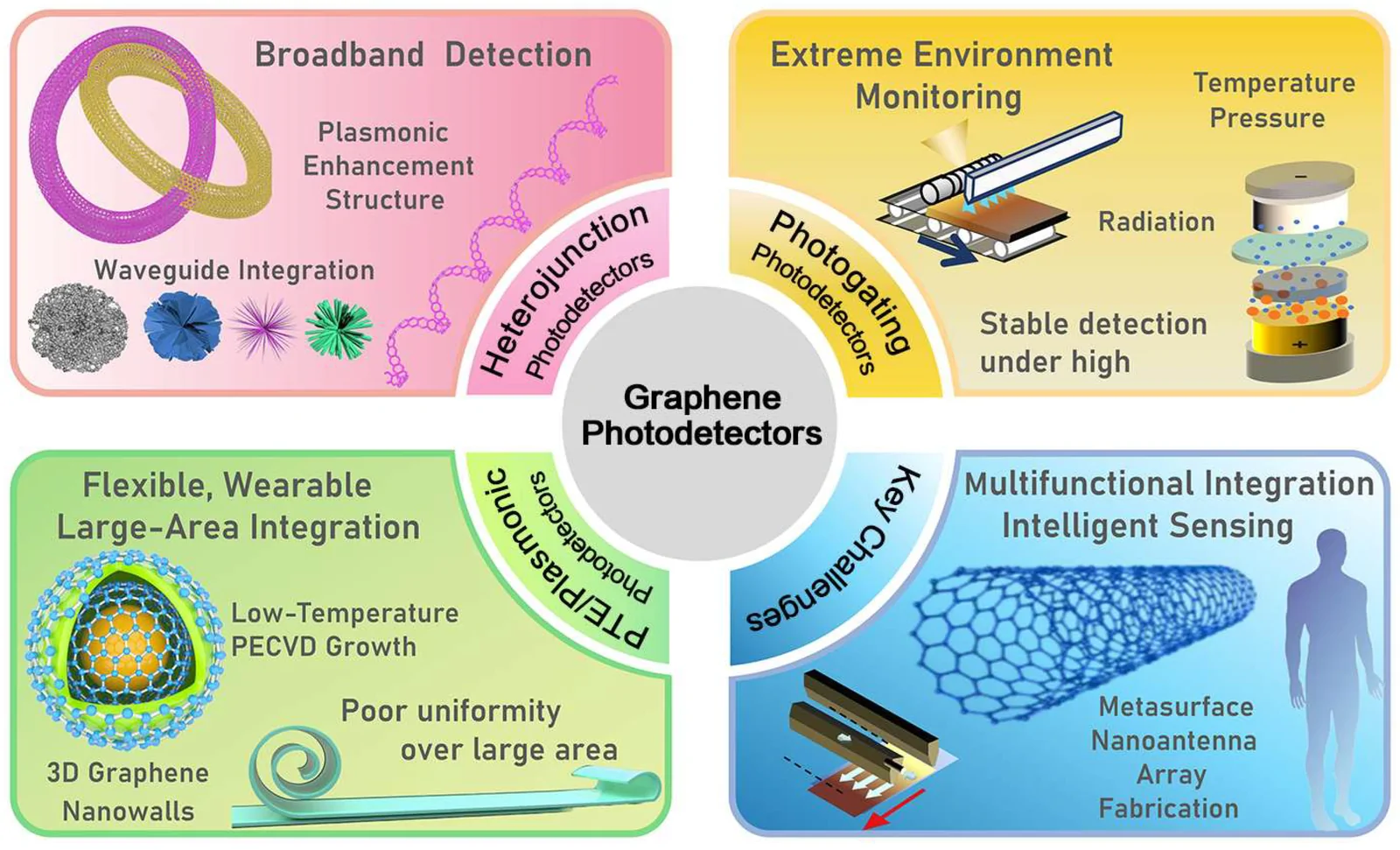
Core device types and application prospects of graphene photodetectors.

**Figure 2:**
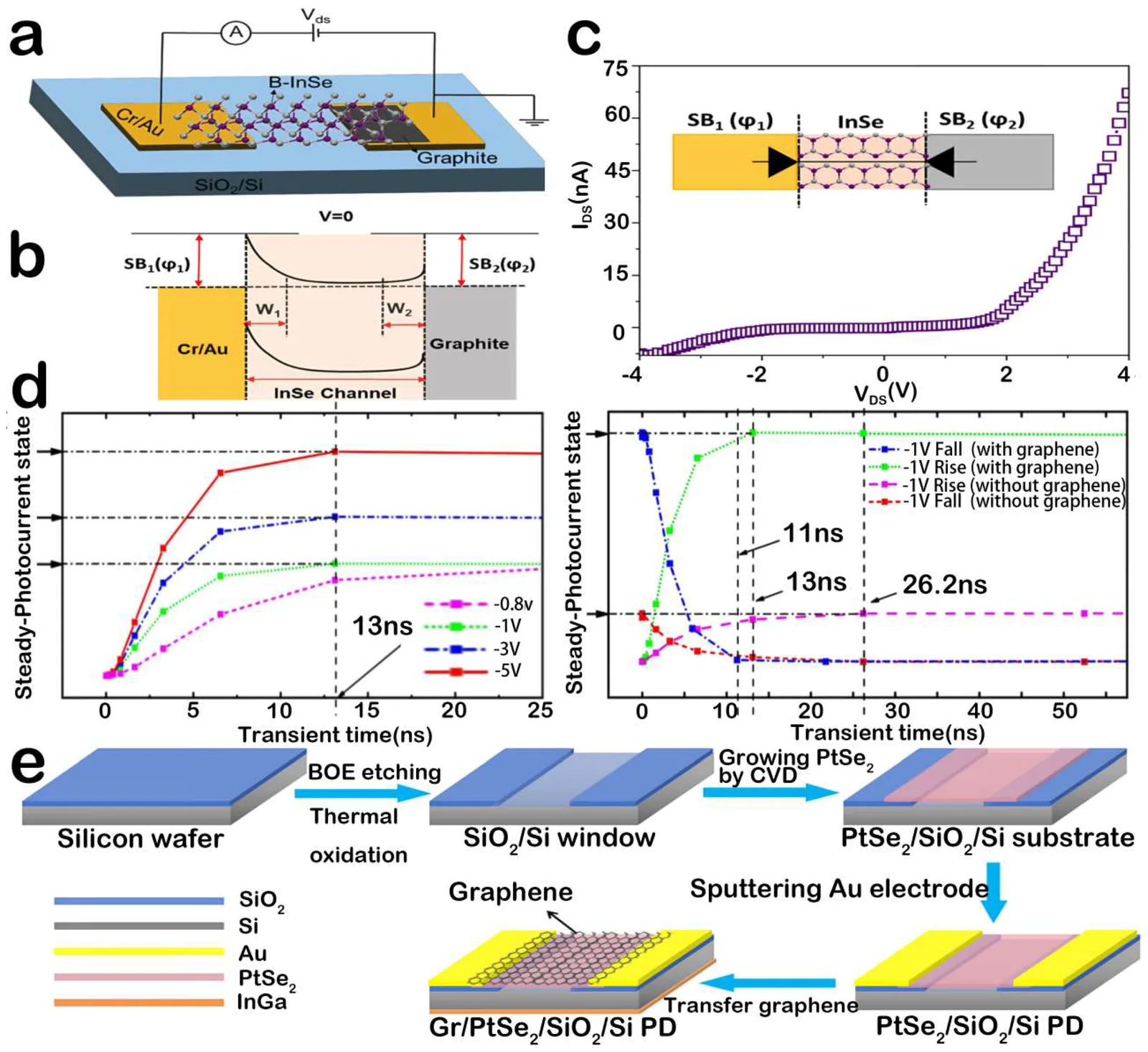
Structural schematic diagrams and performance characteristics of several representative graphene-based photodetectors developed in recent years. (**a**) Three-dimensional structure diagram of the AU-INSE-GR asymmetric heterojunction detector. (**b**) Diagram of the asymmetric metal contact zone of Au-InSe-Gr. (**c**) The characteristic I-V curve of the Au-InSe-Gr diode; the illustration shows the schematic diagram of back-to-back diodes. Graphics copied with the permission of the reference materials [18]. All rights reserved 2025, Royal Society of Chemistry. (**d**) The increase and decrease times of the optical response of graphene/InSb/AlSb heterointegrated devices under different bias voltages and the increase and decrease times of the optical response of device structures with and without graphene. The three horizontal dashed lines in the left panel represent the steady-state photocurrent baselines under different reverse bias voltages (−1 V, −3 V, −5 V). The two horizontal dashed lines in the right panel represent the steady-state photocurrent baselines for the devices with and without graphene at −1 V bias. These baselines serve as reference levels for extracting the rise and fall times. Graphics copied with the permission of the reference materials [20]. All rights reserved 2025, Institute of Electrical and Electronics Engineers Inc. (**e**) Schematic diagrams of the novel two-dimensional semiconductor PtSe_2_/ultrathin SiO_2_/SiO_2_ PD prepared with and without graphene top electrodes. Graphics copied with the permission of the reference materials [22]. All rights reserved 2025, Multidisciplinary Digital Publishing Institute.

**Figure 3:**
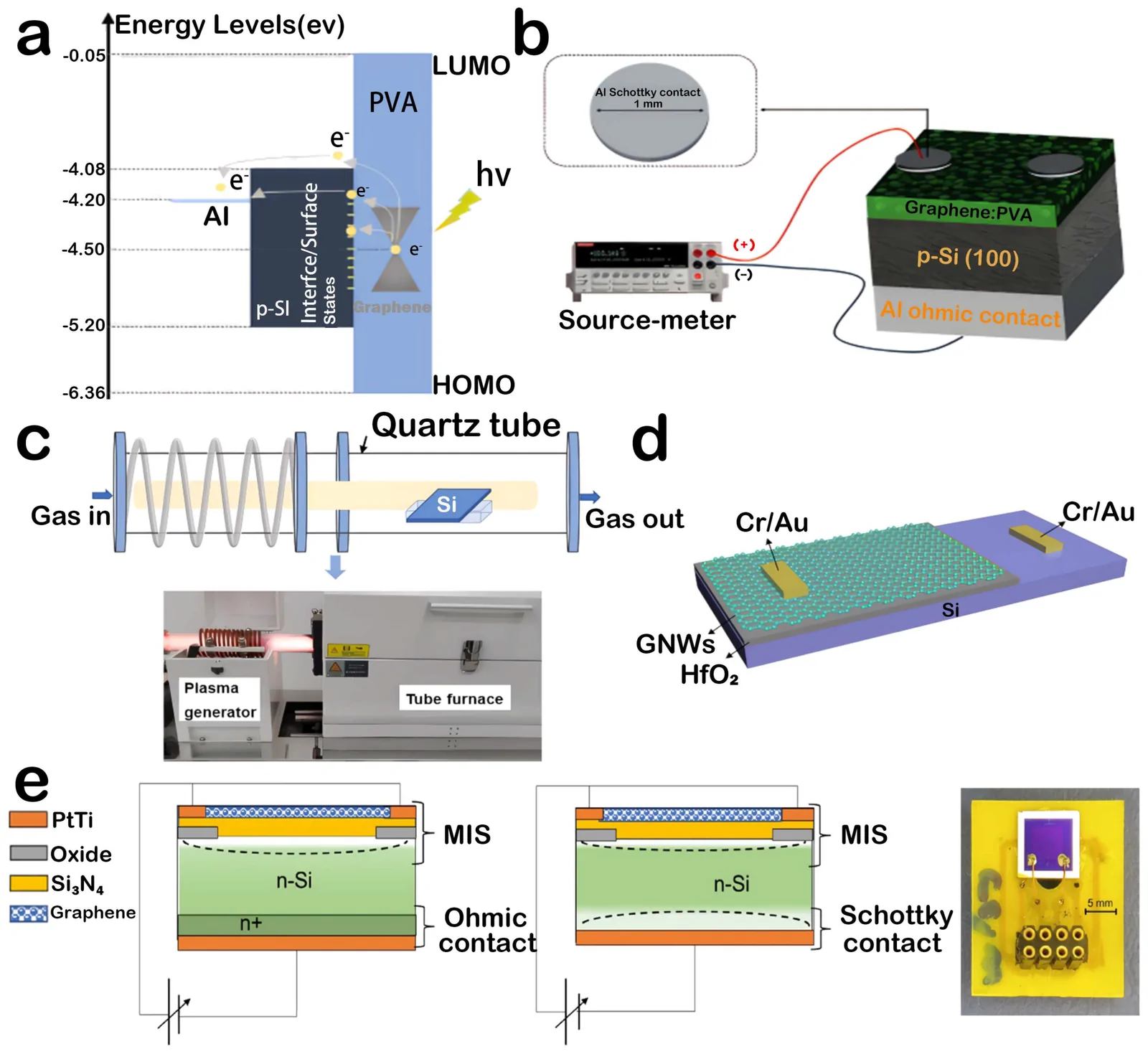
Structure and working mechanism of graphene-based photodetectors. (**a**) PVA/p-Si Schottky structure band diagram. (**b**) Visual effect of the measurement system and the preferred diode. Graphics copied with the permission of the reference materials [25]. All rights reserved 2024, American Chemical Society. (**c**) Schematic diagram and physical photos of the remote radio frequency plasma-enhanced horizontal tube deposition system. (**d**) Schematic diagram of the structure of GNWs/HfO_2_/Si photodetector. Graphics copied with the permission of the reference materials [26]. All rights reserved 2023, Multidisciplinary Digital Publishing Institute. (**e**) Schematic diagram of the device based on heterojunction: SLGr/Si_3_Ni_4_/n-Si (MIS) junction, with (left) ohmic contact and (middle) Schottky contact on the back, (right) optical image of the SLGr/Si_3_Ni_4_/n-Si device. Graphics copied with the permission of the reference materials [31]. All rights reserved 2025, Springer Nature.

**Figure 5:**
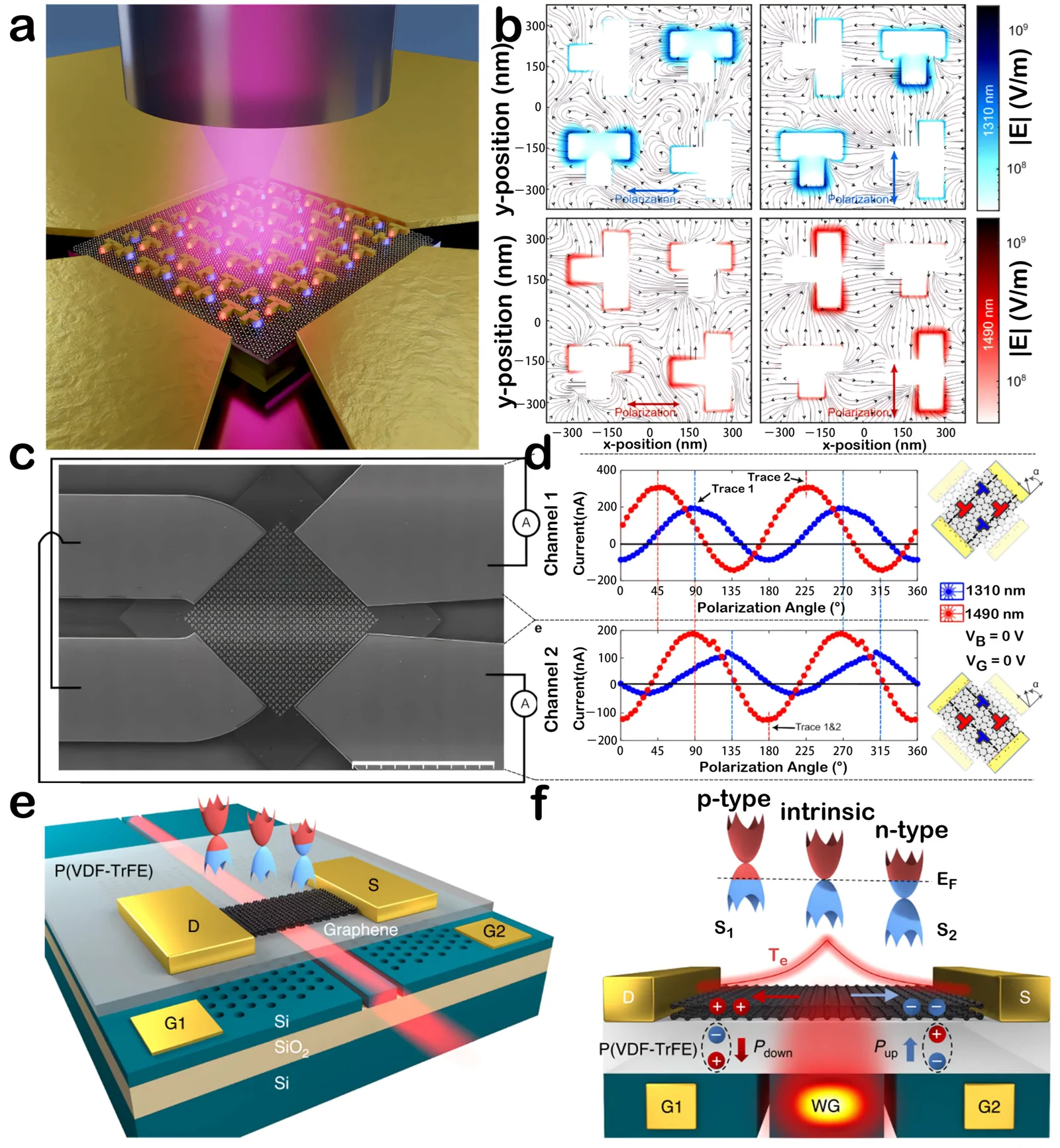
The structure and working mechanism of graphene ultrafast photodetectors. (**a**–**d**) Supercell metamaterials are perfect absorbers and multiplexers. (**a**) Artistic schematic diagram of the structure of a four-contact multi-resonant device. (**b**) Simulation of the absolute electric field response of a single cell under excitation of four different wavelengths and polarizations. (**c**) Scanning electron microscope image of the four-port device. (**d**) Curves of the current response of the two channels under 1310 nm and 1490 nm illumination varying with the polarization angle. Graphics copied with the permission of the reference materials [49]. All rights reserved 2024, Springer Nature. (**e**) Schematic diagram of the graphene p-i-n homojunction photodetector. (**f**) Schematic diagram of the Seebeck coefficient gradient and the temperature distribution of hot carriers on the graphene layer. Graphics copied with the permission of the reference materials [50]. All rights reserved 2025, Springer Nature.

**Figure 7:**
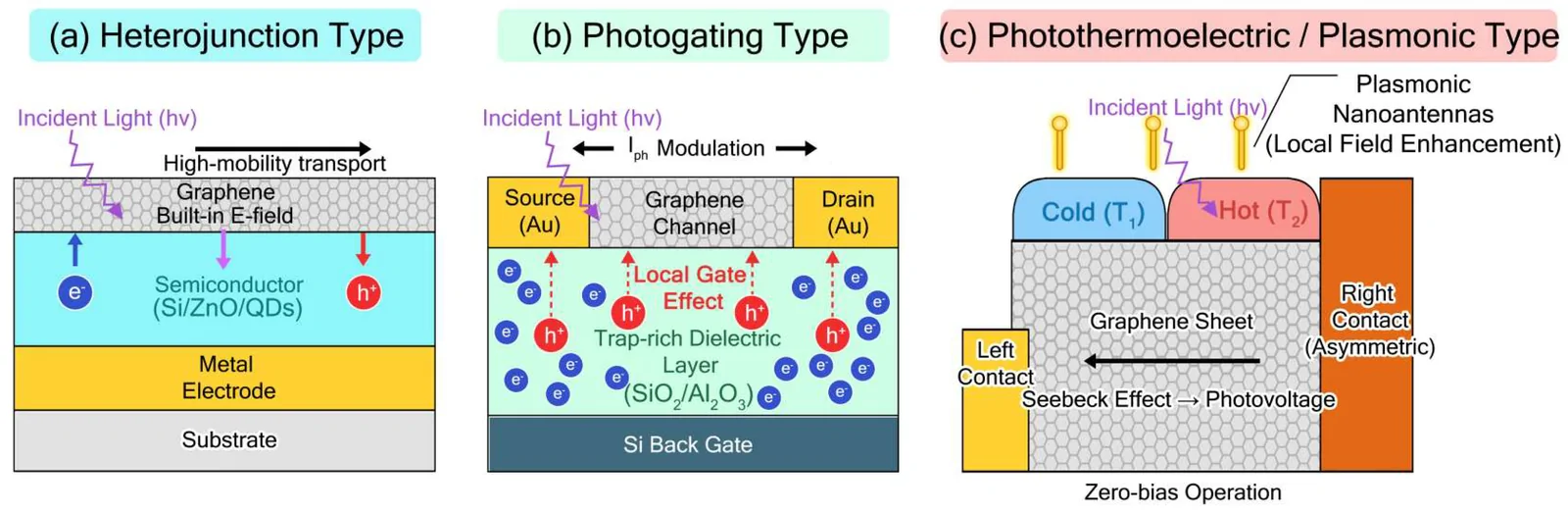
Schematic illustration of three representative graphene-based photodetector architectures. (**a**) Heterojunction type: a vertical graphene/semiconductor/metal stack. The built-in electric field at the interface drives the separation of photogenerated electron-hole pairs, while graphene serves as a high-mobility transport channel. (**b**) Photogating type: a lateral field-effect transistor (FET) structure with a trap-rich dielectric layer. Trapped charges generate a local gate effect, modulating the graphene channel conductance to achieve high photoconductive gain. (**c**) Photothermoelectric/plasmonic type: a planar graphene sheet with asymmetric metal contacts and integrated plasmonic nanoantennas. Light-induced local heating creates a temperature gradient, generating a photovoltage via the Seebeck effect under zero-bias operation.

**Figure 9:**
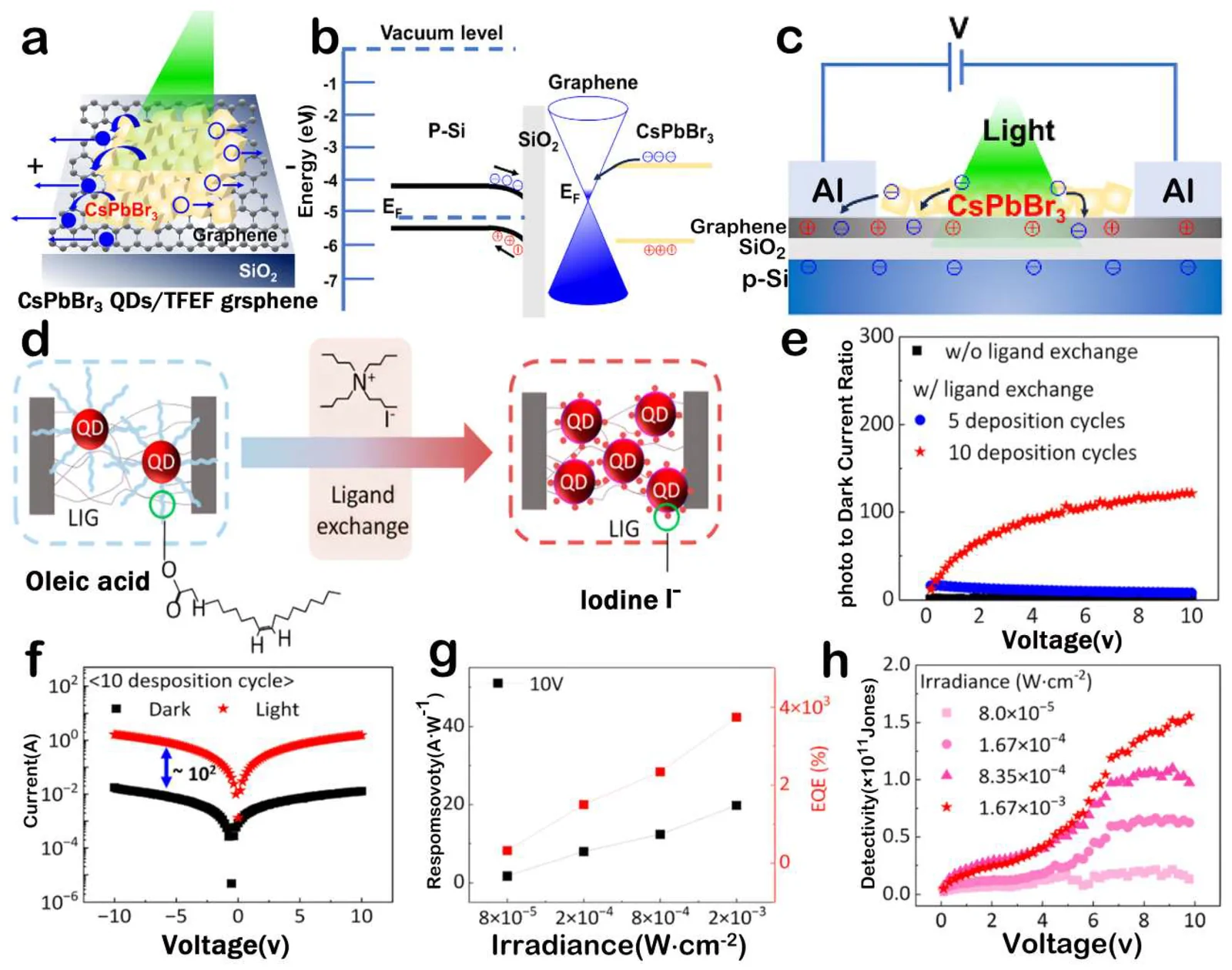
Structural characterization and photoelectric performance of graphene-based quantum dot heterojunction photodetectors. (**a**) Graphene containing TFEF. (**b**) Energy band diagram and (**c**) schematic photoresponse mechanism of the CsPbBr_3_ quantum dots (QDs)/TFEF graphene MSM PD. The probable schematics of carrier transportation of CsPbBr_3_ QD films under light irradiation. Graphics copied with the permission of the reference materials [81]. All rights reserved 2025, IOP Publishing Ltd. (**d**–**h**) Laser-induced graphene and colloidal quantum dot heterojunction photodetector. (**d**) Schematic diagrams of QD-LIG heterostructure photodetectors before and after ligand exchange. (**e**) The light-dark current ratio (PDCR) as a function of applied bias for photodetectors without ligand exchange (w/o, using pristine quantum dots with long-chain oleic acid ligands) and with ligand exchange (w/, using TBAI-treated quantum dots with short iodine ligands) under different quantum dot deposition cycles. (**f**) Current-voltage characteristics under dark and illuminated conditions. (**g**) Responsivity and external quantum efficiency of the photodetector measured at a 10 V bias relative to irradiance. (**h**) The detection rate of photodetectors measured under different irradiance. Graphics copied with the permission of the reference materials [82]. All rights reserved 2025, Royal Society of Chemistry.

**Figure 10:**
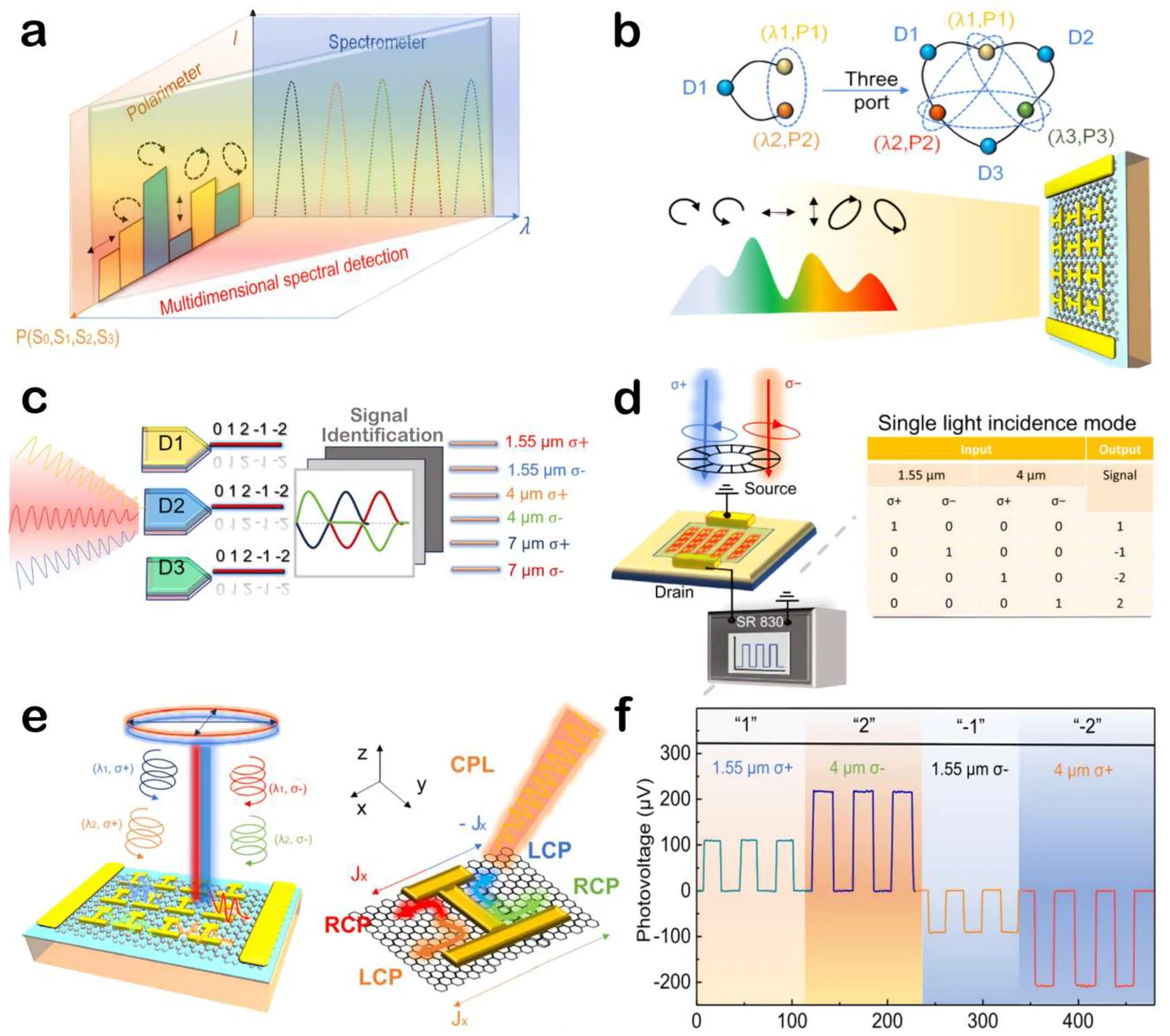
The overall solution for intelligent high-dimensional spectral detection. (**a**) This scheme combines deep learning with multi-dimensional sensors to achieve three-dimensional simultaneous detection. (**b**) Graphene devices modified by nano-antennas generate differentiated photocurrents for light of different wavelengths and polarizations, achieving direct decoupling of wavelength and polarization information. (**c**) Schematic diagram of extracting three-wavelength circularly polarized signals. (**d**) Schematic diagram of the optical information coding device and optical information coding in single light incident mode. (**e**) The dual-arm subsurface structure on graphene can position light of different wavelengths and chirality on both sides of the dual arms, generating vector photocurrents of different magnitudes and directions. (**f**) The encoded optical voltage output signal corresponding to the single light incident mode. Graphics copied with the permission of the reference materials [93]. All rights reserved 2024, Springer Nature.

**Table 1:** Summary of representative works on heterojunction-type graphene photodetectors.

Category	Material/Structure System	Working Mechanism	Detection Band	Key Performance Metrics	Ref.
Low-Dimensional Van der Waals Heterojunction	Graphene/quantum dots	Van der Waals heterojunction	UV-IR	Ultrahigh responsivity potential	[12]
	Au-InSe-Gr	Asymmetric van der Waals heterojunction	Vis-NIR	Dark current: 0.1 nA at −1 V	[18]
	Graphene/ZnO nanowire arrays	1D/2D heterojunction	UV	Ultrafast response	[19]
	Graphene/PtSe_2_/ultra-thin SiO_2_/Si	Van der Waals heterojunction	Broadband	High responsivity, fast response	[22]
3D Bulk Semiconductor Heterojunction	Graphene/Ge	Schottky heterojunction and chemical doping	NIR	Enhanced responsivity, suppressed dark current	[10]
	Graphene/InSb/AlSb	PN heterojunction	NIR	High responsivity and high speed	[20]
	GNWs/HfO_2_/Si	Schottky heterojunction	—	Self-powered	[26]
	Graphene/GaAs	Interface state-induced gain	Vis-NIR	Ultrahigh photoconductive gain	[36]

**Table 2:** Overview of typical photogating-type graphene photodetectors.

Category	Material/Structure System	Working Mechanism	Detection Band	Key Performance Metrics	Ref.
Si-based Photogating	Graphene/SiO_2_/p-Si	Interfacial photogating effect	Vis-NIR	Broadband ultrahigh response	[38]
	Graphene-oxide-Si	Photogating effect	Broadband	Back-gate tunable photocurrent	[39]
	Graphene/PtSi	Photogating high gain	1310 nm	Responsivity: 1.5 A/W	[41]
III-V and 2D Material Photogating	Graphene/InSb	Photogating effect	MIR 4.6 μm	Responsivity enhancement: 1000-fold	[43]
	WSe_2_/Ta_2_NiSe_5_	Photogating-assisted tunneling	—	Response time: µs; high responsivity	[45]
Hybrid/Engineered Photogating	Ionic-polymer-gated graphene	Photogating effect	—	Enhanced responsivity	[40]
	Multi-channel parallel electrode graphene	Structural optimization	—	Enhanced photocurrent	[44]

**Table 3:** Summary of research progress on photothermoelectric/plasmonic-enhanced graphene photodetectors.

Category	Material/Structure System	Working Mechanism	Detection Band	Key Performance Metrics	Ref.
Plasmon-Enhanced Type	Nano-perforated graphene layer	Plasmonic resonance and rectification	THz	Responsivity: 0.10 A/W	[48]
	Graphene/metamaterial absorber	Photothermoelectric and plasmonic	NIR	3 dB bandwidth: up to 400 GHz	[49]
	Graphene/gold fractal metamaterials	Plasmonic resonance	Broadband	Responsivity enhancement: approximately 100-fold	[52]
Waveguide-Integrated Type	Graphene p-i-n/ferroelectric dielectric/Si waveguide	Photothermoelectric effect	Telecom	Responsivity: 193 mA/W; bandwidth: 17 GHz	[50]
	Graphene waveguide-integrated	Plasmonic enhancement	Telecom	Responsivity: 3.72 A/W; NEP: 4.54 pW/$\sqrt{Hz}$	[51]
Photothermoelectric-Dominant Type	Flexible graphene/PET	Photothermoelectric effect	UV-mmWave	Ultra-broadband response	[54]
	3D graphene foam	Photothermoelectric effect	UV-microwave	Responsivity: 10^3^ A/W	[55]
	Graphene/metasurface	Photothermoelectric effect	MIR	Polarization detection	[56]

**Table 4:** Comparative analysis of heterojunction, photogating, and photothermoelectric/plasmonic graphene photodetectors.

Device Type	Core Mechanism	Key Advantages	Key Limitations	Typical Applications	Ref.
Heterojunction Type	Built-in electric field at graphene/semiconductor interface drives carrier separation	Broadband detection; self-powered operation possible; high responsivity; versatile material combinations	Gain-speed trade-off; wet transfer affects uniformity/yield; interface stability environment-dependent	Broadband imaging; optical communication; multispectral detection	[7,8,9,10,11,12,13,14,15,16,17,18,19,20,21,22,23,24,25,26,27,28,29,30,31,32,33,34,35,36]
Photogating Type	Trapped photogenerated carriers modulate graphene channel conductance via capacitive coupling	Ultra-high photoelectric gain; ideal for weak-light sensing; simple device architecture	Gain-speed trade-off; saturation at high light intensity; narrow dynamic range; interface drift affects stability	Low-light detection; infrared sensing; on-chip spectral sensing	[37,38,39,40,41,42,43,44,45,46,47]
Photothermoelectric/Plasmonic Type	Temperature gradient drives carrier diffusion or plasmonic resonance enhances light absorption	Zero-bias operation; ultrafast response; low power consumption; polarization sensitivity	Low intrinsic absorption; thermal scattering limits conversion efficiency; complex nanofabrication requires high consistency	High-speed communication; flexible/wearable devices; terahertz detection	[48,49,50,51,52,53,54,55,56,57,58,59]

**Table 5:** Application cases of graphene photodetectors in extreme environment monitoring.

Category	Application Scenario	Core Contribution	Key Technology	Ref.
High/Low Temperature Environments	High-temperature sensing (300–575 K)	TCR temperature sensor	CVD single-layer graphene	[61]
	High-temperature detection (700 °C)	TCR temperature sensor	Full encapsulation technology	[62]
	Ultra-broadband detection (183–501 K)	Bi_2_Se_3_-PtSe_2_ heterojunction	Multi-physical effect integration	[69]
High-Pressure/Mechanical Damage Environments	High-pressure detection (6.4 GPa)	High-pressure-induced phase transition	Diamond-like carbon phase	[63]
	Mechanical damage environment	Graphene/self-healing polymer	Self-healing composite material	[67]
UV/Solar-Blind Environments	Deep-UV detection (500 K)	Graphene/4H-SiC MSM	Graphene transparent electrode	[64]
	Solar-blind UV detection	rGO/AlGaN	Thermally reduced GO electrode	[65]
	High-temp solar-blind UV and neuromorphic computing (610 K)	High-temperature UV detector	Optical signal encryption processing	[74]
Corrosive/Complex Environments	Corrosion-resistant flexible detection	Zr-doped amorphous Ga_2_O_3_	Amorphous oxide	[73]
	Adaptive multi-mode detection	VO_2_/MoTe_2_ heterojunction	Phase-change material integration	[75]

**Table 6:** Graphene photodetectors for flexible/wearable applications and large-scale integration.

Category	Application Direction	Core Contribution	Key Technology	Refs.
Flexible Wearable Detection	Flexible ultra-broadband detection	PET/graphene and asymmetric electrodes	Zero-bias operation	[51]
	Flexible PbS-graphene	PI in situ grown graphene	Repeated bending stability	[73]
Green/Low-Temperature Preparation	Eco-friendly graphene/CsPbBr_3_ QDs	Natural camphor carbon source and transfer-free	Low-cost green preparation	[78]
	Vitamin C-reduced rGO	Natural camphor carbon source and transfer-free	Conductivity restoration	[81]
	PVA-modified rGO	Polymer modification	Low-power detection	[82]
Wafer-Level Large-Scale Integration	Wafer-level graphene/Ge	Wafer-level integration	High-performance Schottky junction	[75]
	200 mm wafer graphene-QD hybrid	Full-process wafer-level technology	Imaging sensor array	[76]
	Laser-induced graphene/QDs	LIG direct patterning	Customizable flexible devices	[79]

**Table 7:** Representative works on graphene photodetectors for multifunctional integration and intelligent sensing.

Category	Application Direction	Core Contribution	Key Technology	Refs.
Waveguide-Integrated Type	Waveguide-integrated detection	Dual-core waveguide and microring resonator	High responsivity, low NEP	[87]
	Thin-film lithium niobate waveguide integration	Graphene/TFLN platform	High-speed detection and electro-optic modulation	[89]
	Waveguide-integrated optoelectronic devices	Waveguide-integrated graphene photonic devices	Sensing and communication applications	[95]
Plasmonic/Metasurface-Integrated Type	Metasurface-assisted high-dimensional detection	Nano-antenna array and graphene	Direct wavelength/polarization decoupling	[93]
	Plasmonic-assisted integrated detection	Bias-free plasmonic enhancement	Integrated graphene detectors	[94]
Heterogeneous Integration Type	Dual-mode detection	Graphene/InP van der Waals heterojunction	Photovoltaic/photoconductive mode switching	[88]
	Graphene transparent electrode enhancement	Graphene/NiO/Si heterojunction	Enhanced carrier collection efficiency	[90]
	Graphene nanoribbon/IGZO heterojunction	1D nanomaterial heterojunction	Self-powered broadband detection	[91]

**Table 8:** Summary of full-band detection capabilities of graphene photodetectors.

Category	Material/Structure System	Working Mechanism	Detection Band	Key Performance Metrics	Ref.
UV–Deep UV Detection	Gr/i-GaN/n-GaN p-i-n structure	Heterojunction	340 nm	Responsivity: 20.6 A/W	[97]
	Graphene/Si (HfO_2_ dielectric layer)	MIS structure	120–200 nm	EQE: approaching/exceeding 100%	[100]
Visible–NIR Detection	Graphene/PbS heterojunction	Heterojunction	265–2200 nm	Responsivity: 72 A/W at 792 nm	[101]
	Graphene/GaAs heterojunction	Heterojunction	808 nm	Responsivity: 122 mA/W	[104]
FIR–THz Detection	Quasi-free-standing bilayer graphene and antenna	Photothermoelectric effect and antenna matching	THz	Responsivity: 35 V/W; NEP: less than 300 pW/$\sqrt{Hz}$	[109]
	Graphene/black phosphorus–black arsenic heterojunction	Interband detection	FIR (6–12 μm)	Responsivity: 3.68–10.08 A/W	[110]

## Data Availability

No new data were created or analyzed in this study. Data sharing is not applicable to this article.

## Abbreviations

The following abbreviations are used in this manuscript:

BP	Black Phosphorus
CMOS	Complementary Metal-Oxide-Semiconductor
D*	Specific Detectivity
EQE	External Quantum Efficiency
FET	Field-Effect Transistor
h-BN	Hexagonal Boron Nitride
IR	Infrared
MIS	Metal-Insulator-Semiconductor
MSM	Metal-Semiconductor-Metal
NEP	Noise Equivalent Power
PECVD	Plasma-Enhanced Chemical Vapor Deposition
TCAD	Technology Computer-Aided Design
TCR	Temperature Coefficient of Resistance
UV	Ultraviolet
